# Development and experimental validation of a composite rod underwater acoustic wireless charging system

**DOI:** 10.1371/journal.pone.0355236

**Published:** 2026-08-20

**Authors:** Sichen Zou, Zeyu Si, Wei Sun, Shiquan Ma

**Affiliations:** 1 Laoshan Laboratory, Qingdao, PR China; 2 Beihai Research Station, Institute of Acoustics, Chinese Academy of Sciences, Qingdao, PR China; 3 Naval Submarine Academy, Qingdao, PR China; Guangdong University of Petrochemical Technology, CHINA

## Abstract

With the increasing application demands of unmanned underwater vehicles in various marine exploration missions, underwater energy supply has become one of the major bottlenecks restricting their performance enhancement and application expansion. Traditional battery-powered methods are limited by endurance and operational duration, failing to meet the requirements for long-term and continuous operation. As an innovative energy transmission approach, underwater acoustic wireless charging technology leverages the propagation characteristics of sound waves in water to provide a non-contact, long-distance wireless charging solution. This paper investigates key technologies in underwater acoustic wireless charging, including the design and fabrication of high-power, highly directional transducer arrays for transmission and reception. To address the energy needs of underwater vehicles for prolonged continuous operation, this study proposes a prototype development plan for a long-distance underwater acoustic wireless charging system and conducts relevant validation tests in a anechoic tank environment.

## Introduction

One of the primary constraints on enhancing the performance and expanding the applications of unmanned underwater vehicles is the issue of underwater energy supply. Traditional underwater charging technologies predominantly rely on wet-mateable connectors. However, these connectors are constrained by lengthy seabed cables, which entail exorbitant application costs. Moreover, wet-mateable connectors impose stringent requirements on pressure resistance and sealing performance. During operation, they are prone to wear, susceptible to seawater corrosion, and consequently exhibit short service lives along with compromised safety and reliability [1].

Currently, research on underwater wireless charging technology primarily focuses on the principle of electromagnetic induction [2–5]. To address load variations, reference [6] designed an underwater wireless power transfer system capable of maximizing transmission efficiency. In reference [7], a magnetic resonant wireless power transfer system for AUVs, comprising three ferrite-core coils with a diameter of 2 meters each, was proposed, achieving an efficiency of 77–80%. However, challenges such as electromagnetic wave attenuation [8,9] interference from irregular ocean currents [10], and imprecise underwater docking accuracy [11] pose significant difficulties for the practical application of electromagnetic induction-based wireless charging technologies.

In contrast, underwater acoustic wireless charging technology utilizes sound waves as the energy carrier. As a form of mechanical wave, sound waves exhibit low propagation attenuation, high directivity, and are unaffected by the electrical conductivity of the medium in seawater. Consequently, they typically achieve longer operating distances, exceeding one meter. Reference [12] presents an experimental study on ultrasonic wireless energy transfer, achieving milliwatt-level power reception over a distance of one meter. Reference [13] confirms the feasibility of wirelessly powering underwater sensors using acoustic sources through analytical models. Reference [14] develops an ultrasonic wireless energy transfer method for the underwater Internet of Things, while Reference [15] proposes a novel high-power underwater wireless power transfer scheme based on an acoustic transducer array. Nevertheless, current research on underwater wireless charging via acoustic waves remains largely in the stages of principle validation and scheme design. Its practical application potential and feasibility in real marine environments still require further exploration.

Addressing the energy supply challenges for unmanned underwater vehicles and sensor networks, this study focuses on breakthroughs in key technologies for high-power underwater acoustic wireless charging. A prototype system capable of achieving long-distance and relatively high-power wireless energy transmission in aqueous media has been successfully developed. The system performance was validated through tank testing. Although the system exhibits relatively low electro-acoustic conversion efficiency, this limitation is compensated by the minimal acoustic propagation attenuation in water, demonstrating significant advantages in transmission distance.

### System composititon

**Fig 1:**
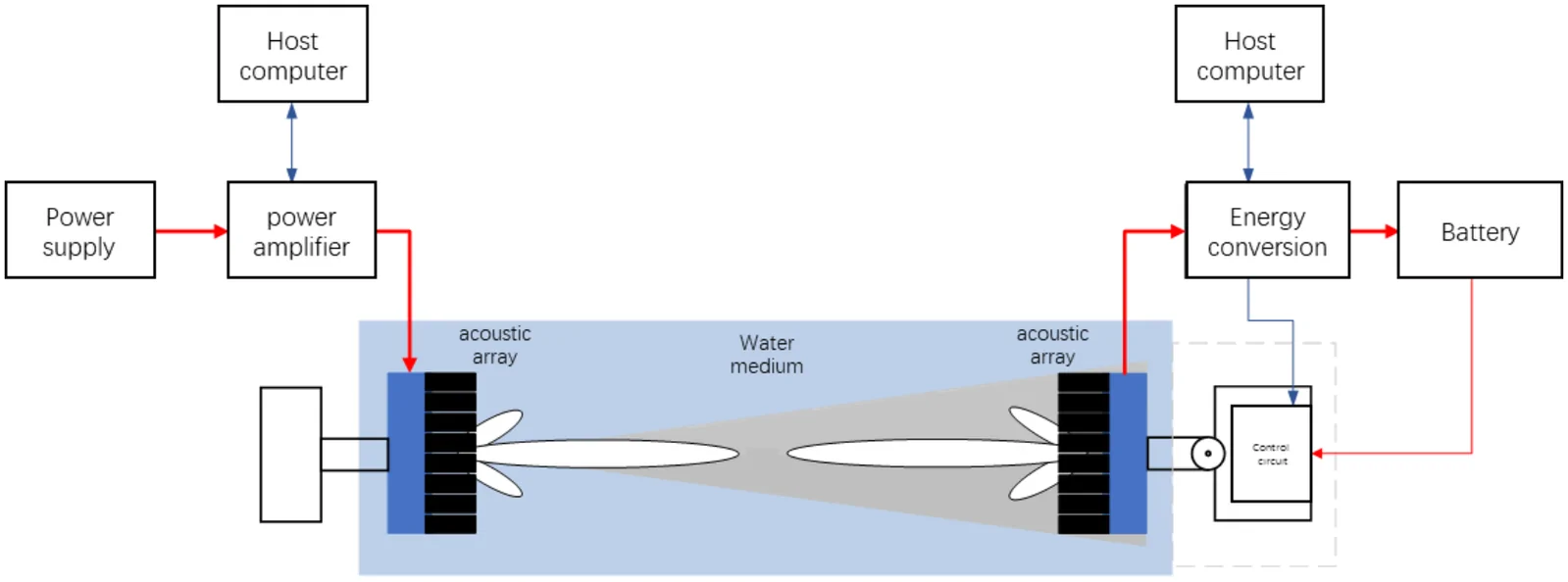
System composition.

The composite piezoelectric transducer-based underwater acoustic wireless charging system primarily consists of a composite piezoelectric transmitter and receiver transducer array along with energy conversion circuitry, as illustrated in Fig 1.

The composite rod transducer-based acoustic transmission and reception array primarily consists of transducer elements and corresponding array structures. It enables highly efficient electro-acoustic or acoustic-electric energy conversion at specific resonant frequencies, while emitting or receiving acoustic energy with sharp directivity. Research on high-power acoustic projectors is relatively mature, with common technical solutions including cylindrical transducers, flextensional transducers, and disc transducers. However, these solutions generally exhibit insufficient directivity, leading to significant acoustic divergence over long distances, making them unsuitable for underwater acoustic charging applications. The primary technical approach for achieving highly directional transmission is through large-scale projector arrays composed of numerous densely arranged transducer elements.

Utilizing a high-power, highly directional projector array to transmit acoustic energy requires a high-power amplifier circuit capable of driving the transmitting array effectively. Similarly, to efficiently utilize or store the energy received by the acoustic receiver array, an optimal conditioning circuit must be implemented between the receiving array and the load.

### System design

#### Structural design of the composite rod trancducer.

**Fig 2:**
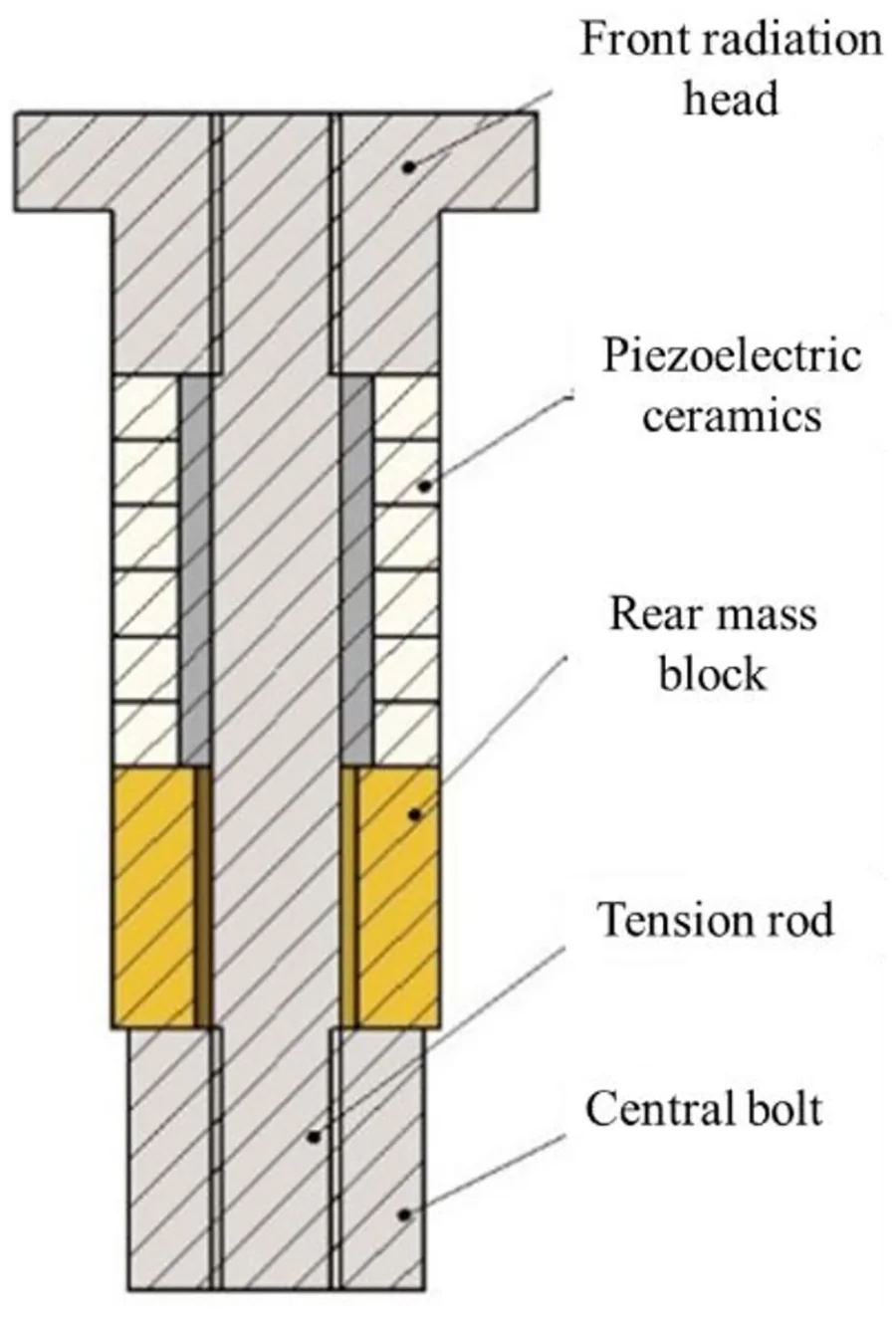
Structure of the composite rod transducer.

The composite rod transducer comprises five main components: piezoelectric ceramics, a front radiation head, a rear mass block, a central bolt, and a tension rod, as illustrated in Fig 2.

**Fig 3:**
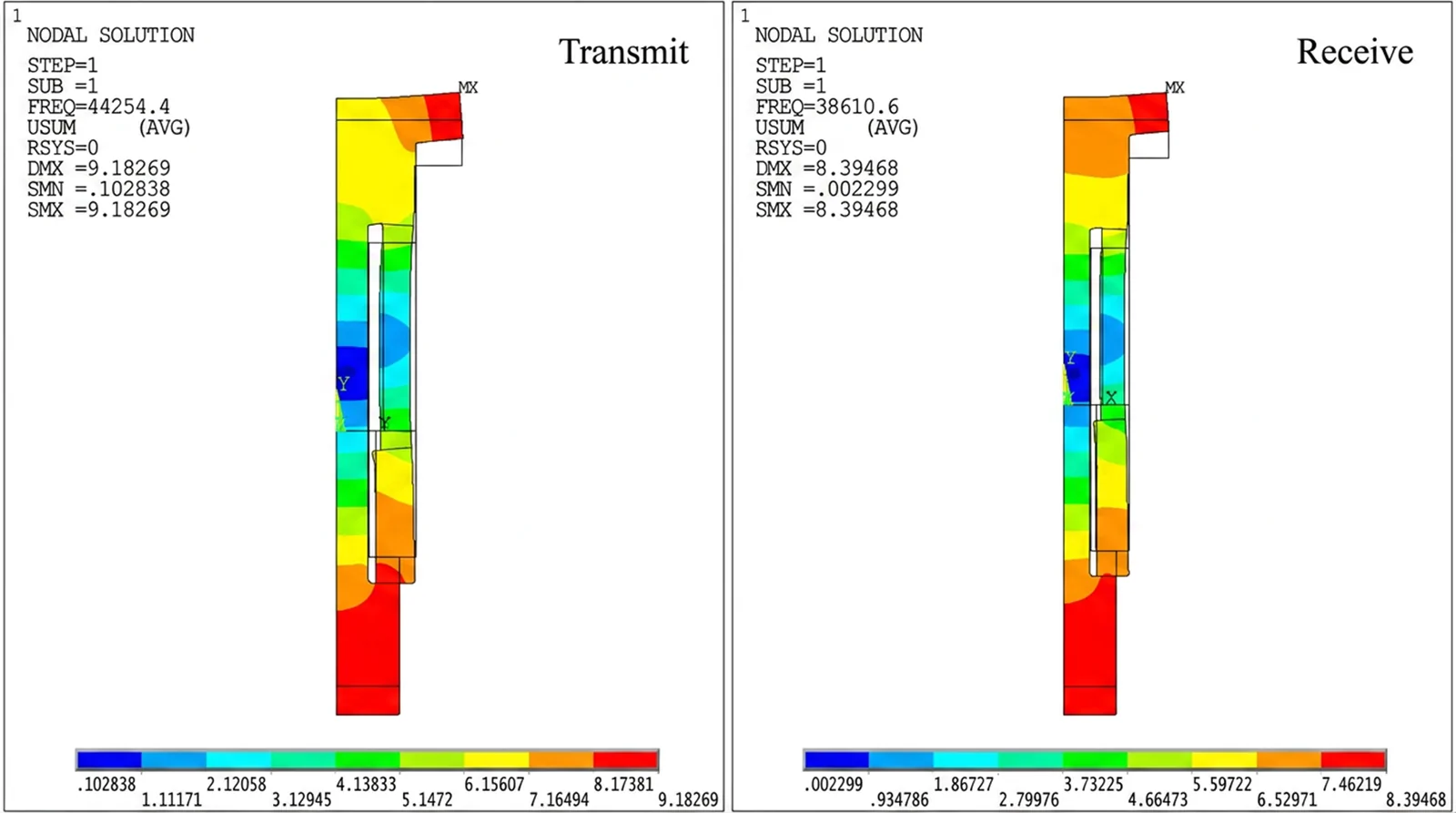
Transmit (left) and receive (right) resonant modes of the transducer, coloured by nodal displacement amplitude.

Both the transmitter and receiver employ a composite rod transducer structure. Since the peak receiving sensitivity occurs at a frequency beyond the peak transmission response frequency, equalizing the optimal frequency of the receiver response with that of the transmitter response ensures maximum energy transfer efficiency. Each transducer element has dimensions of Φ16 mm × 33.5 mm. A finite element model has been established. Fig 3 illustrates the resonant mode of the transducer.

**Fig 4:**
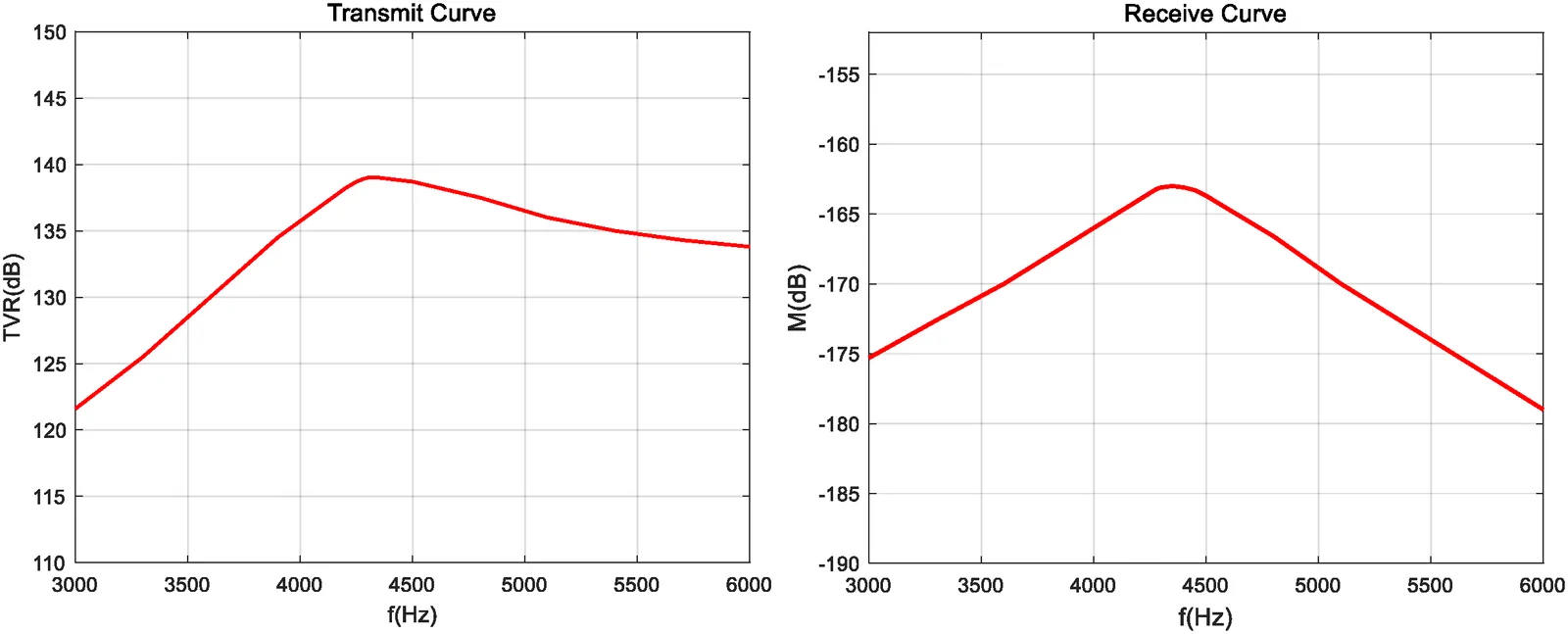
Transmit (left) and receive (right) frequency responses of the transducer over 3–6 kHz.

The frequency response characteristics of the transducer are shown in Fig 4.

It can be observed that the transmitting transducer element achieves its peak transmitting voltage response of 139 dB at 43.5 kHz. At this same frequency, the receiving transducer element exhibits a receiving sensitivity of −163 dB, which coincides with the peak transmitting voltage response frequency.

**Fig 5:**
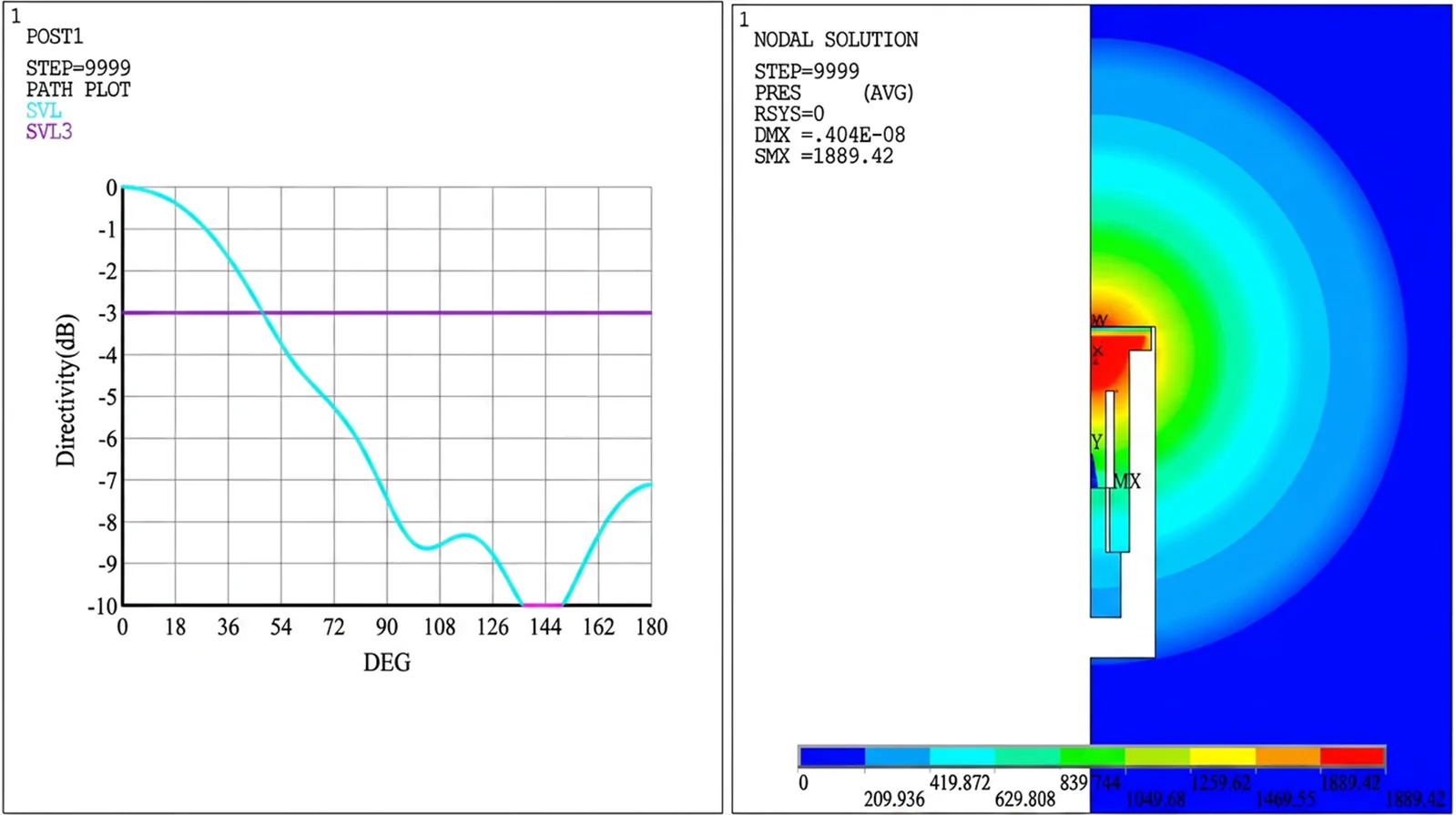
Directivity pattern (left) and near-field sound pressure contour (right) of the transmitting transducer element.

Additionally, a directivity simulation was conducted for the transmitting transducer in Fig 5. The -3dB radiation beamwidth of a single transmitting element is approximately 96°, and a schematic diagram of its underwater directivity beamwidth and radiated acoustic field is shown in the figure below. Since the radiating surface area of the transmitting element is identical to that of the receiving element, according to the principle of acoustic reciprocity, the transmission directivity of the transmitting element is the same as the reception directivity of the receiving element at the same operating frequency.

**Fig 6:**
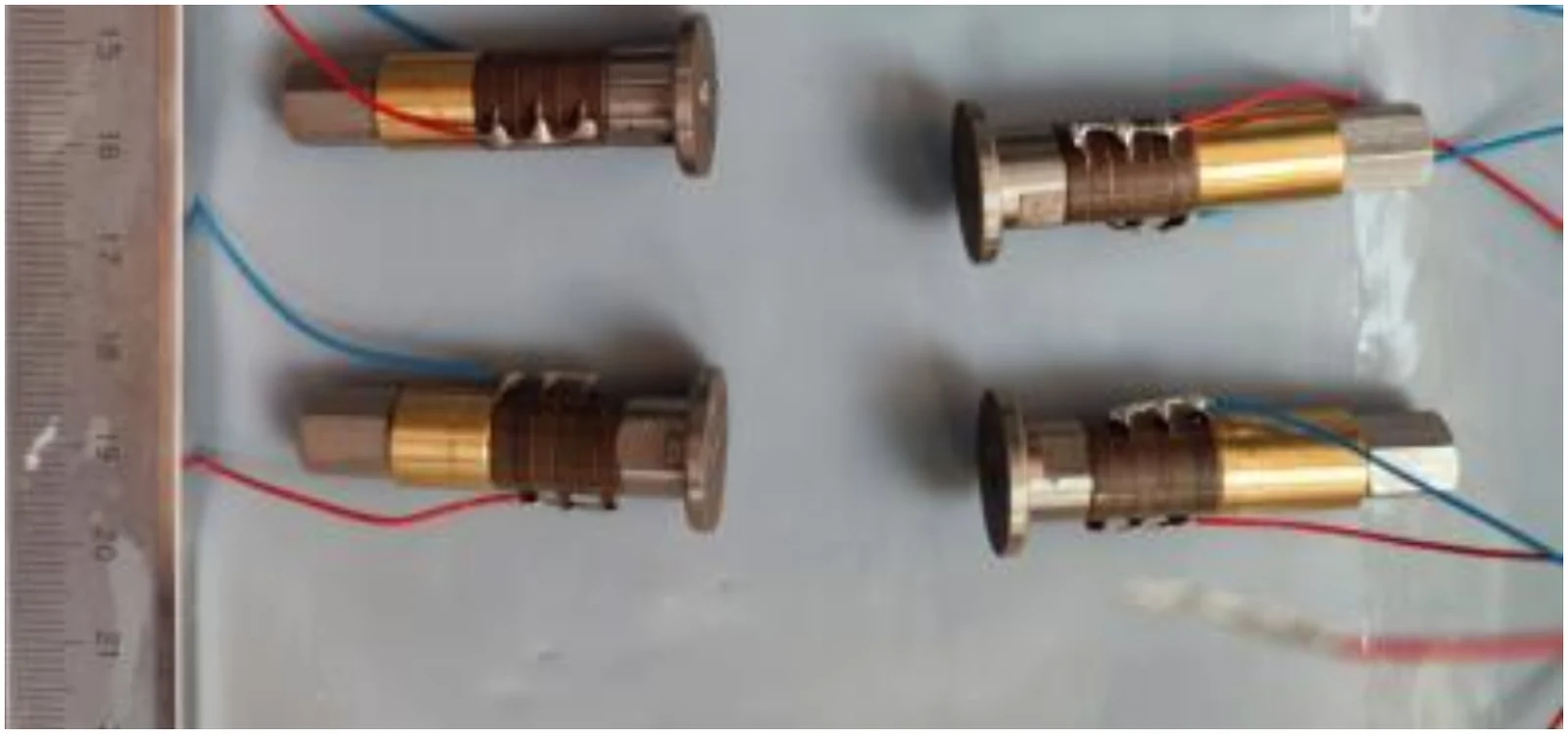
Transducer element.

Prototype units of the transmitting and receiving transducer elements have been fabricated according to the design, as shown in the Fig 6.

### Array design

The number of array elements is estimated based on the source level using the following formula.


$$\begin{array}{c} SL=TVR+\text{2}\text{0}\text{log}\left(V_{rms}\right)+\text{2}\text{0}log(N) \end{array}$$ (1)

Where SL denotes the source level of the acoustic array, TVR represents the transmitting voltage response of a single element, $V_{rms}$ is the root-mean-square excitation voltage applied to the transducer, and N is the number of array elements. Substituting the measured performance of the prototype element into the above formula, it is estimated that an array of 80 elements, with a preset excitation voltage of 200 $V_{rms}$, can achieve a source level of 220 dB.

According to the directivity composite formula for a linear array.


$$\begin{array}{c} R(\theta )=D(\theta )\cdot Y(\theta ) \end{array}$$ (2)

D(θ) represents the directivity of a linear array composed of point sound sources, Y(θ) denotes the directivity of a single array element, and R(θ) signifies the unilateral directivity of the combined array.


$$\begin{array}{c} D(\theta )=\frac{sin\left[\frac{sin(\theta )\pi (a+\text{2}r)N}{\lambda }\right]}{N\cdot sin\left[\frac{sin(\theta )\pi (a+\text{2}r)N}{\lambda }\right]} \end{array}$$ (3)

$$\begin{array}{c} Y(\theta )=\left|\frac{\text{2}J_{\text{1}}(kr\cdot sin(\theta ))}{kr\cdot sin(\theta )}\right| \end{array}$$ (4)

Where, a is the inter-element spacing, r is the radiation surface radius of an array element. N is the number of array elements along a single side, λ is the wavelength of the acoustic wave at the given frequency, $J_{\text{1}}(\theta )$ is the first-order Bessel function, k is the wavenumber at the acoustic frequency, defined as k=2πf/c, where f is the acoustic frequency and c is the speed of sound.

**Fig 7:**
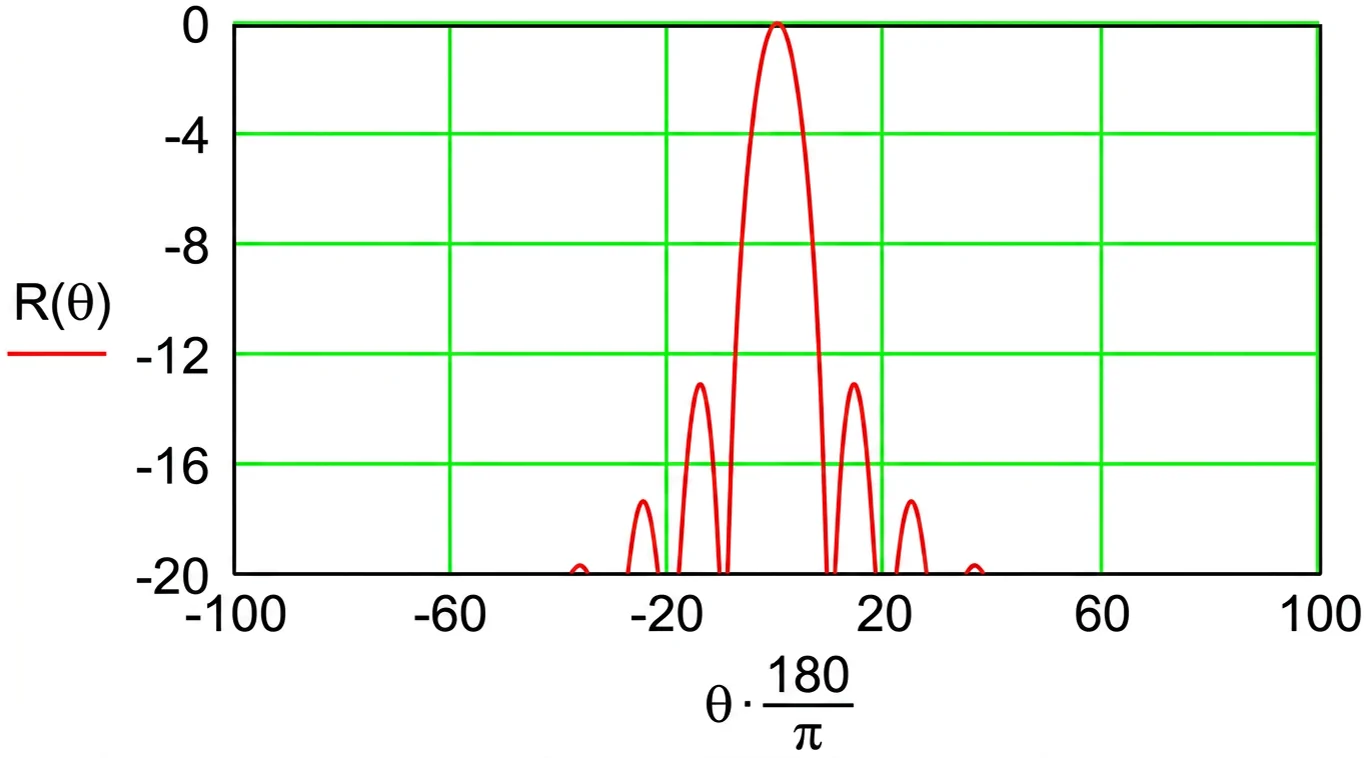
Directivity simulation diagram of the combined array (43 kHz).

The array is designed in a 9 × 9 square configuration with an inter-element spacing of 19 mm. The core section of the complete transducer array measures 204 mm × 204 mm. The transducer array is designed with a −3 dB beamwidth of 8.6°. The simulated directivity diagram of the composite array is shown in Fig 7.

**Fig 8:**
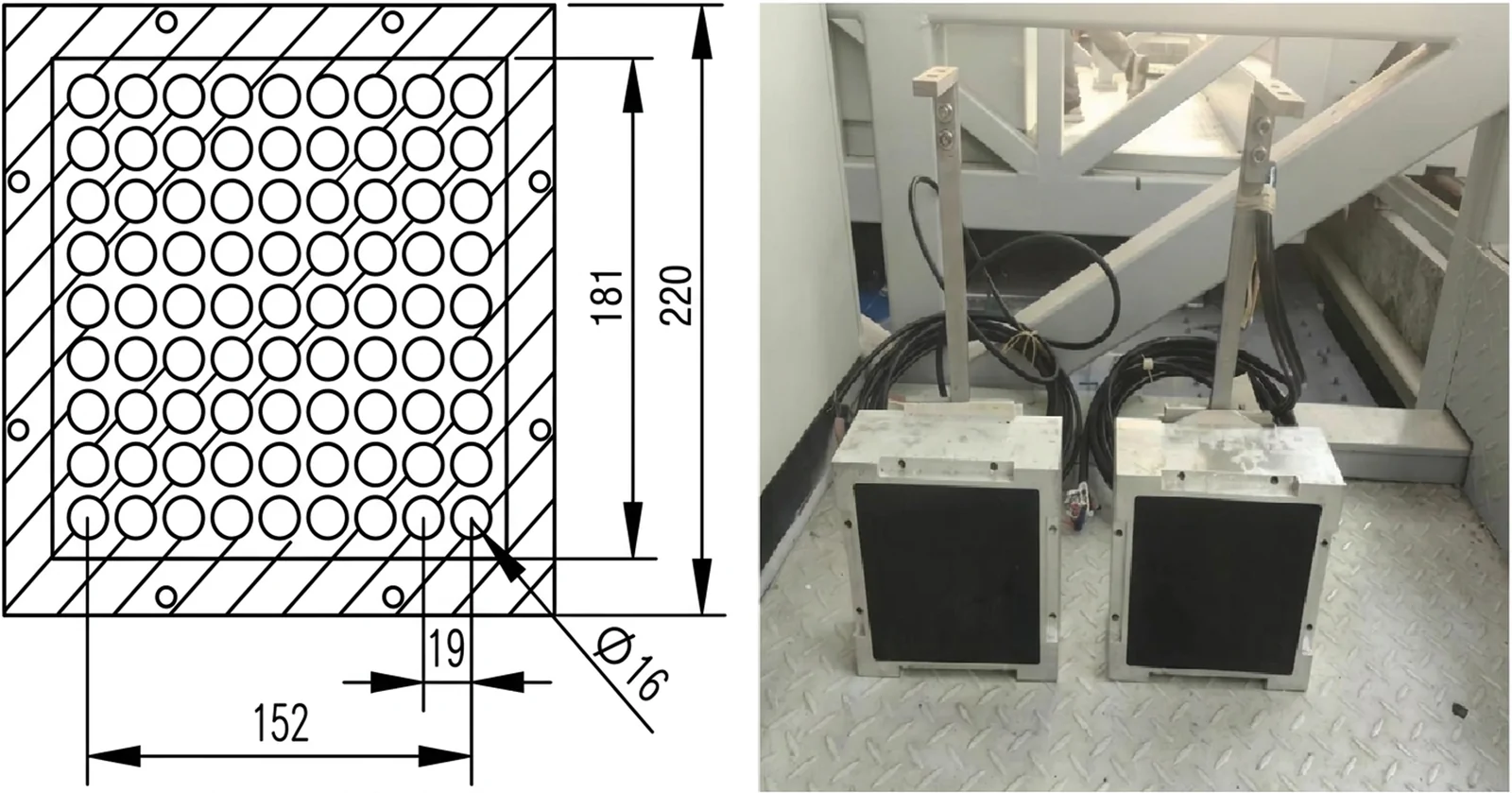
Structure (left) and physical prototype (right) of the transducer array.

The array backing is constructed from rigid polyurethane foam, the housing is made of aluminum alloy, and the radiation surface is encapsulated with waterproof rubber. The internal element wiring adopts a modular 3 × 3 soldering configuration to ensure operational feasibility and connection accuracy. The overall dimensions are 220 mm × 220 mm × 89 mm, with a weight of 8.9 kg in air. Transducer array is shown in Fig 8.

Energy conversion circuit design

**Fig 9:**
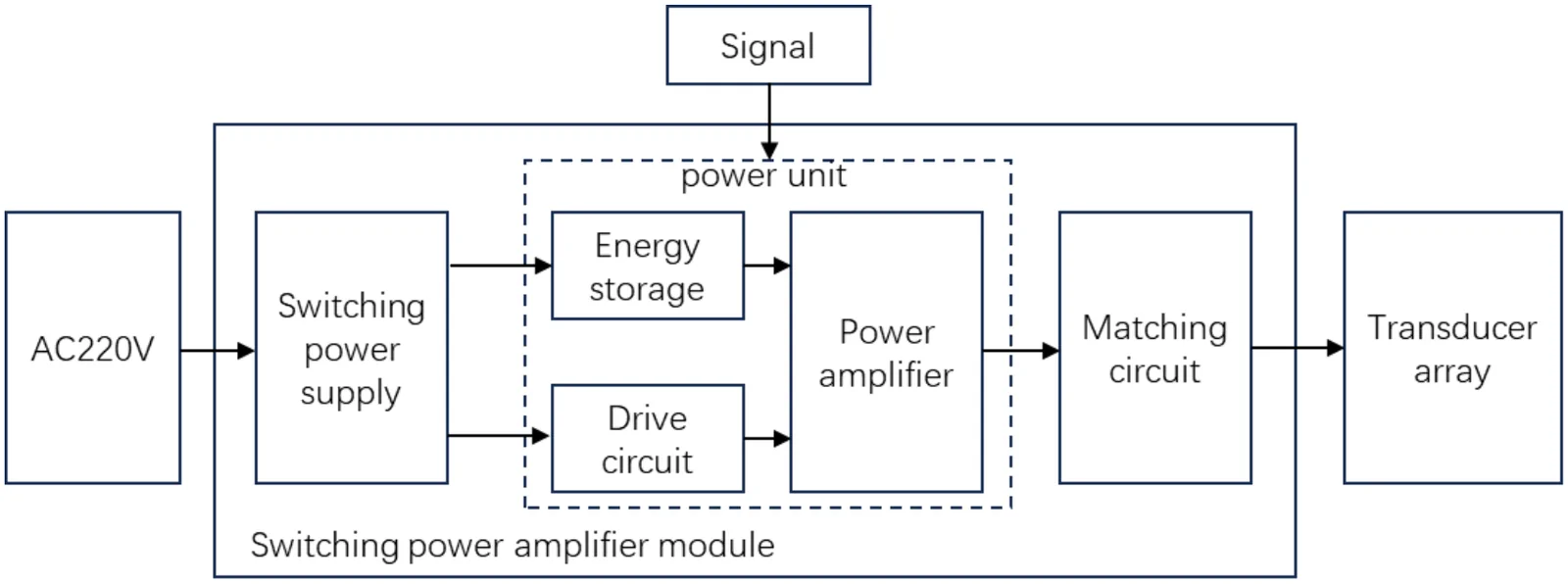
Structure of the power amplifier.

The power amplifier unit consists of three main sections: a switching power supply, a switching power amplifier, and a voltage-boost matching circuit. The switching power supply provides a DC power source ranging from 0 to 200 V to the switching power amplifier. The amplifier module then boosts the input signal and outputs it to the voltage-boost matching circuit, where it is filtered, boosted, and matched to form a sinusoidal waveform that drives the transducer. The internal functional block diagram is shown in Fig 9.

The switching power amplifier operates on a principle similar to that of a Class D power amplifier, with a key distinction: it does not perform signal conversion. The input is a square wave signal at the original frequency, and the output remains a square wave at the same frequency. The output frequency varies with the frequency of the input signal, while the output power depends on the alternating conduction of the internal MOS transistors, enabling adjustable power output.

**Fig 10:**
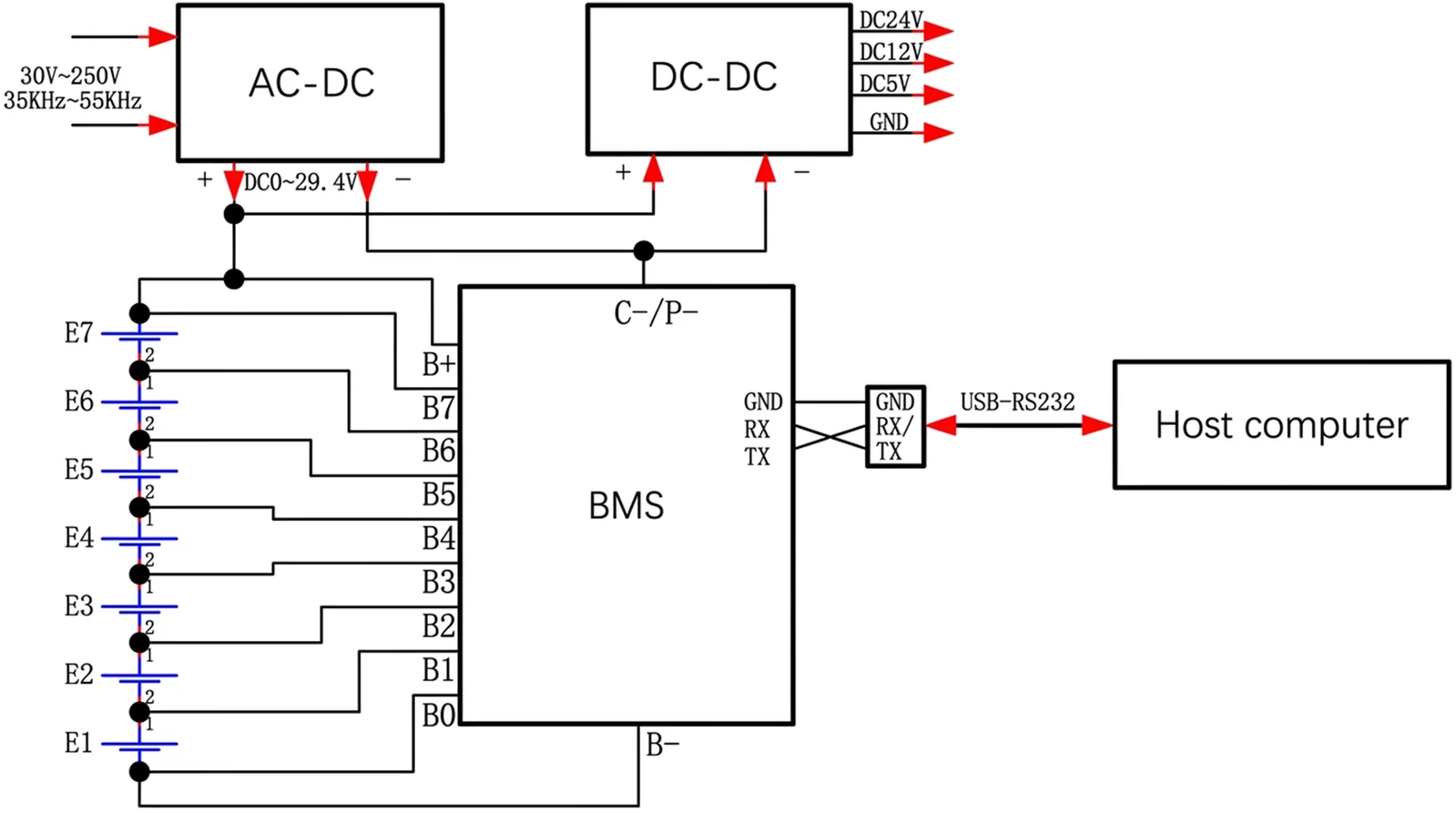
Schematic diagram of the energy harvesting and storage system.

The energy reception and storage system comprises an AC-DC conversion module, a Battery Management System (BMS) module, a DC-DC conversion module, and a battery pack, as illustrated in Fig 10.

Among these, the AC-DC circuit module is used to convert the high-frequency voltage source output by the acoustic receiving array into a stable DC power supply. It can also provide constant current and constant voltage outputs for charging the battery pack.

The BMS includes a control unit, monitoring unit, communication unit, and execution unit. The primary task of the BMS is to ensure safety while maximizing the performance of the battery under various environmental conditions and operational scenarios, thereby avoiding energy waste. Generally, the BMS can be divided into three parts: the controller module, the power interface module, and the sensing chipset. The controller module collects and analyzes information from the battery pack obtained by the sensing chips, processes the data, provides feedback to the BMS, and executes control commands sent by the BMS to the battery pack. It serves as the core of the BMS, acting as the bridge connecting the battery pack and facilitating system communication. The sensing chipset primarily includes sensors for voltage, temperature, etc., responsible for monitoring the operating status of the battery and feeding this information back to the controller.

The DC-DC circuit module is used to convert the battery pack’s output voltage into stable 24 V power supplies for the load. The battery pack serves to store and release electrical energy, ensuring a continuous power supply.

### Anechoic-water-tank experiment

#### Acoustic performance of the transducers array.

**Fig 11:**
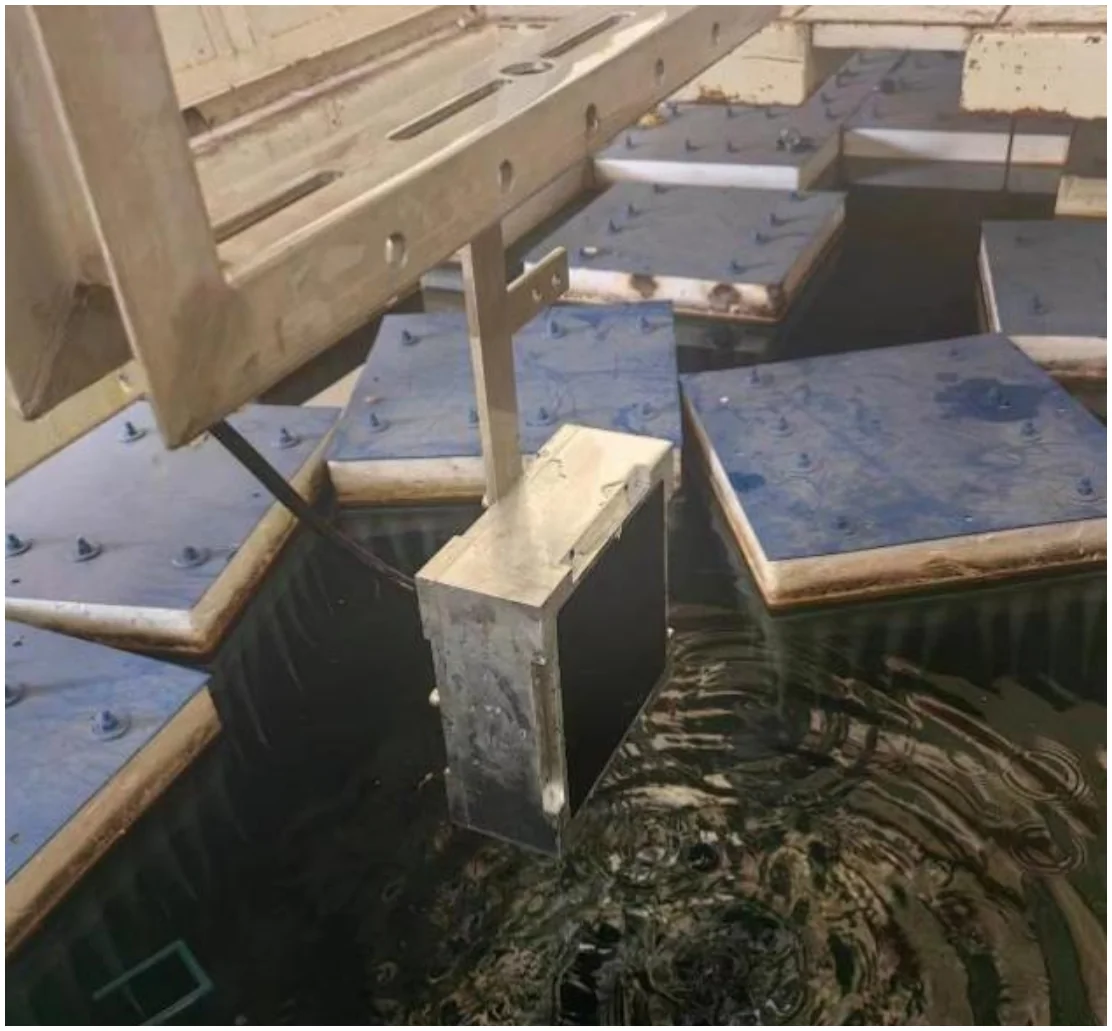
Acoustic performance of the transducers array.

The initial prototype transducers performance testing in an anechoic tank (20m × 12m × 8m). Tests were conducted on two modules 1# and 2# to measure their transmitting voltage response, receiving sensitivity, impedance in water, and directivity patterns, as shown in the Fig 11.

For module 1#, the peak transmitting voltage response occurs at 40.5 kHz with a maximum value of 177 dB, while the peak receiving sensitivity is observed at 46 kHz with a maximum value of −177 dB. The −3 dB beamwidth at 40 kHz measures 10.3°.

**Fig 12:**
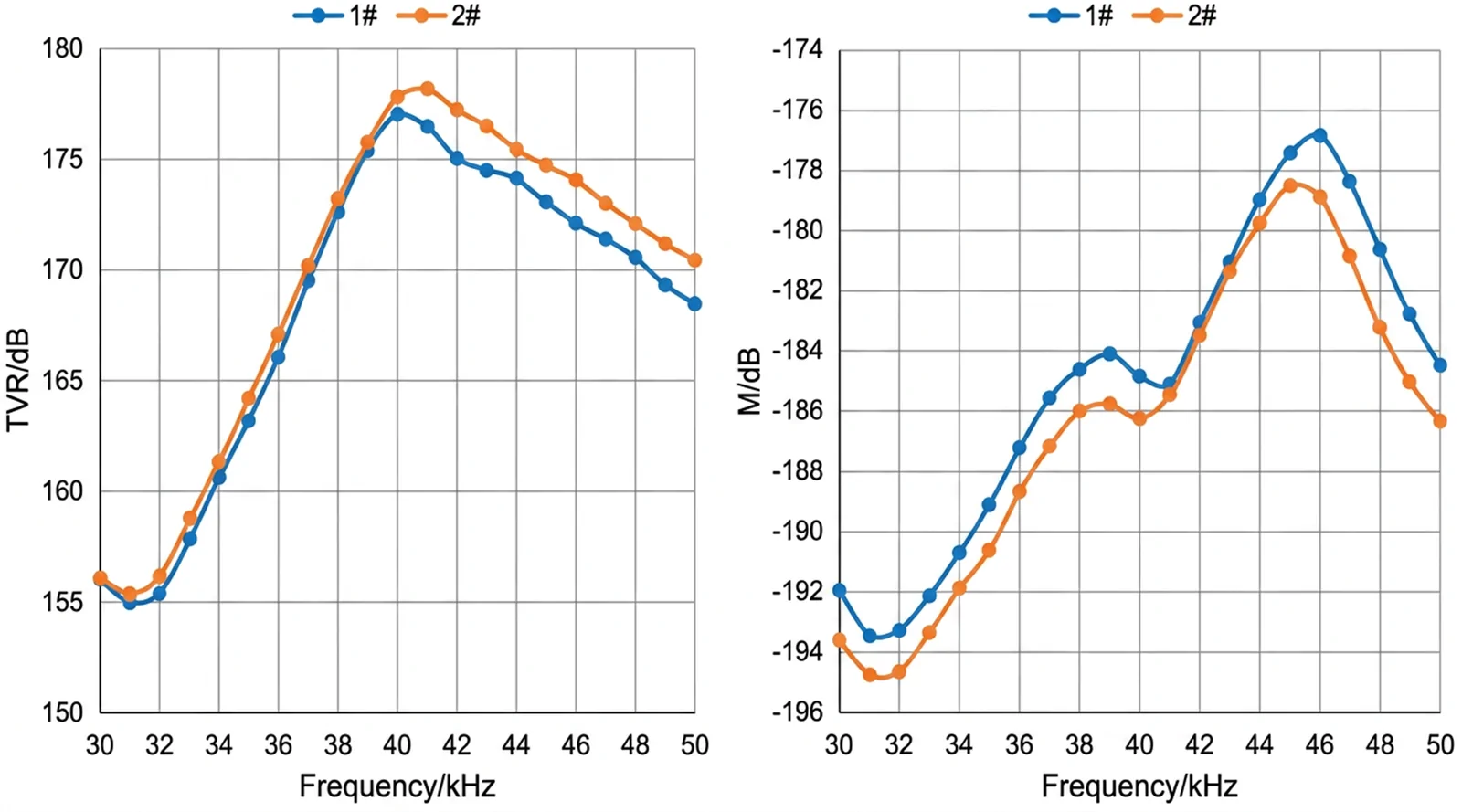
Transmit (left) and receive (right) frequency responses of the transducer for module 1# and 2#.

**Fig 13:**
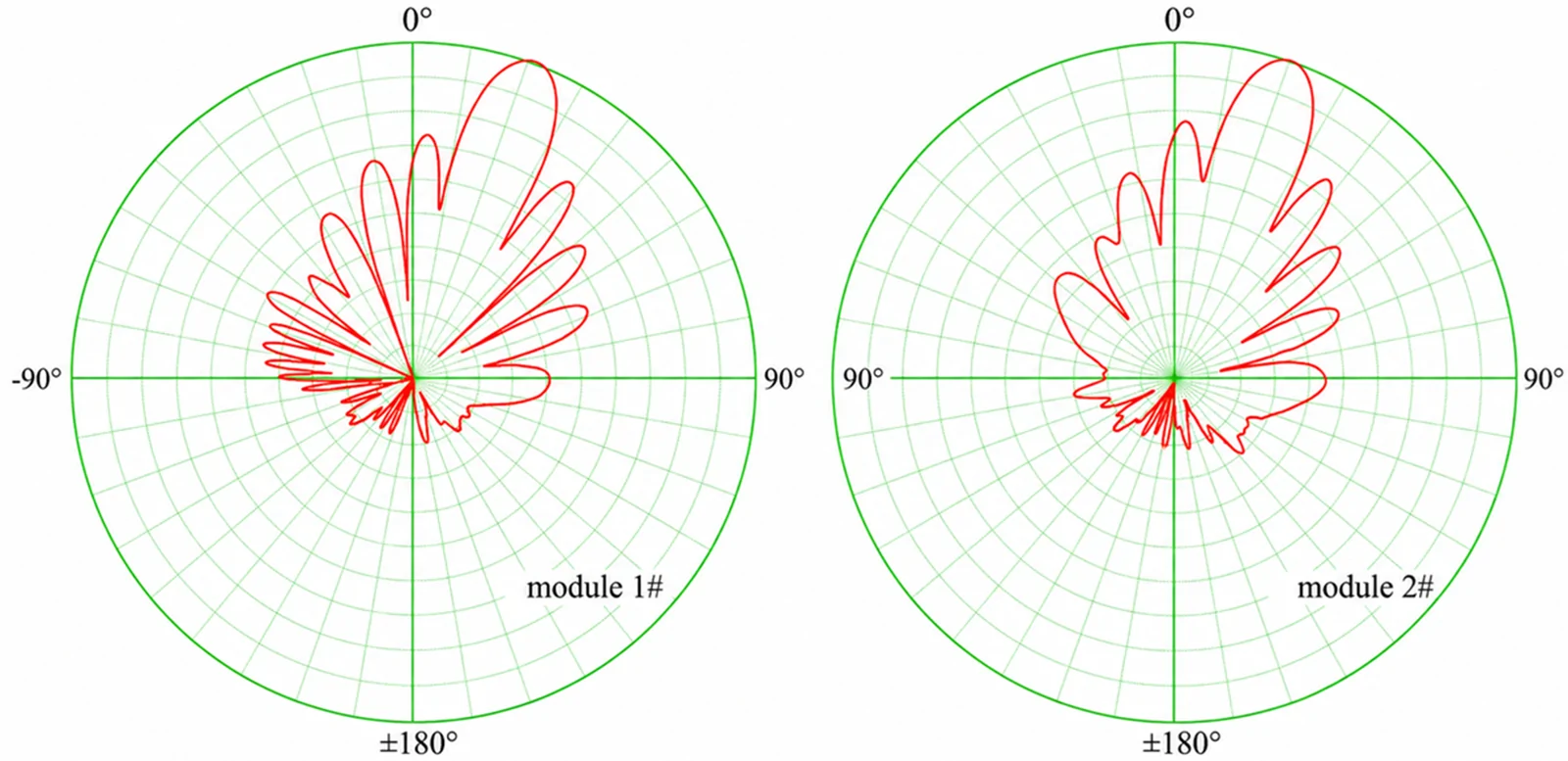
Directivity patterns of two transducer array modules measured at 41 kHz.

For module 2#, the peak transmitting voltage response is located at 41 kHz with a maximum value of 178 dB, and the peak receiving sensitivity appears at 45.5 kHz with a maximum value of −178.5 dB. The −3 dB beamwidth at 40 kHz measures 10.6°. The frequency response characteristics of the transducers array are shown in Fig 12. The directivity of the transducers array are shown in Fig 13.

Due to minor inconsistencies in element assembly and the fact that the testing environment was not an ideal free-field condition, there are slight discrepancies between the measured acoustic performance indicators of the two transducers and the design parameters. However, these deviations remain within acceptable tolerances, indicating that the test results are consistent with the simulation outcomes.

### charging performance

Following the acoustic performance tests of the transducers, a wireless charging experiment was conducted using the two transducer arrays. module 2# was configured as the transmitting array, while module 1# served as the receiving array.

According to acoustic theory, a transducer must operate under far-field conditions to accurately reflect its acoustic performance. The far-field criterion is given by:


$$\begin{array}{c} d\geq \frac{a^{\text{2}}}{\lambda } \end{array}$$ (5)

Where d is the transmitter–receiver distance, a is the maximum aperture of the transducer, and λ is the acoustic wavelength at the operating frequency.

When the receiver is located in the near-field region ($d<a^{\text{2}}/\lambda$), the acoustic field lies in the Fresnel interference zone. In this region, the superposition of acoustic waves is significant, and the sound pressure distribution oscillates drastically with both distance and lateral position, no longer following the spherical wave attenuation law valid under far-field conditions. Further reducing the distance does not monotonically improve the charging efficiency; on the contrary, due to destructive interference, the efficiency may exhibit local troughs and poor spatial repeatability. Consequently, efficiency measurements obtained in the near-field region cannot represent the true acoustic output capability of the transducer itself and are unsuitable for evaluating the performance of wireless power transfer systems.

Based on the physical mechanisms described above, this work conducts experiments under far-field distances to ensure the physical significance and reproducibility of the results. Both transducers were mounted on a deployment frame with a horizontal separation of approximately 1.03 meters.

A signal generator produces a sinusoidal waveform, which is amplified by a power amplifier to drive the transmitting transducer, generating a high-energy acoustic wave signal. The receiving transducer is connected to a purely resistive sliding rheostat. Upon receiving the acoustic signal, the transducer converts it into an electrical signal, which is simultaneously applied across the terminals of the sliding rheostat. Voltage and current sampling probes are used to monitor the voltage across and the current through the rheostat.

**Fig 14:**
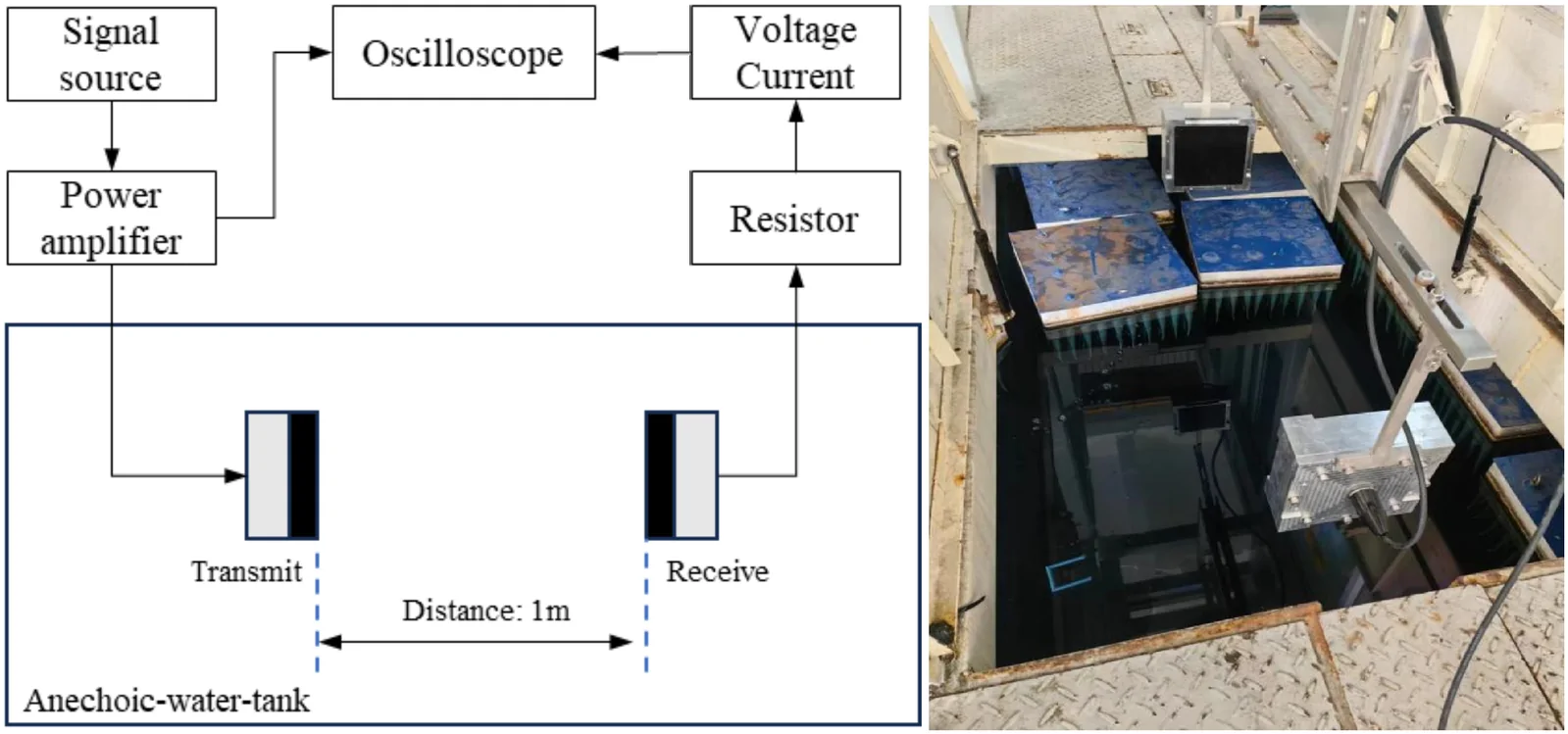
Experimental setup for acoustic wireless charging test.

According to electrical circuit principles, the receiving transducer can be modeled as a power source, and the sliding rheostat as the load. Maximum power is delivered to the load when its resistance matches the internal impedance of the power source. Therefore, during the experiment, the resistance of the sliding rheostat was adjusted based on the measured impedance of the receiving transducer at the operating frequency. The charging experiment is shown in Fig 14.

Based on the distribution of the transducer’s resonance and anti-resonance peaks, the frequency range of 40–45 kHz was selected for the wireless charging performance tests. In accordance with the impedance curve of module 1# and the principle that output efficiency is maximized when internal resistance equals the external load resistance, experiments were conducted by pairing different external load resistances with each frequency point.

**Fig 15:**
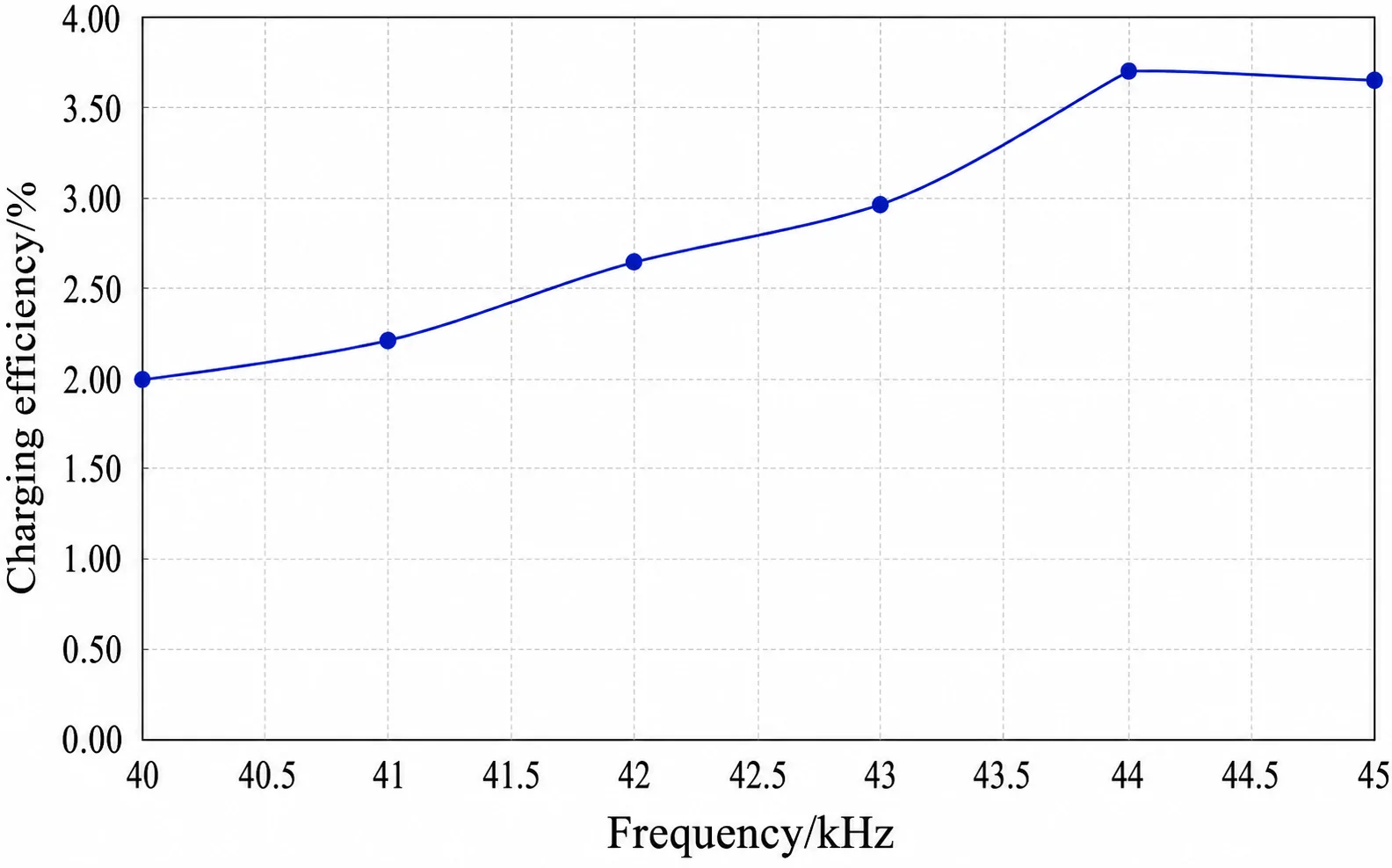
Charging efficiency at different frequency.

The experimental results indicate that the system achieved its highest charging efficiency of 3.71% at a transmission frequency of 44 kHz, where the internal resistance of the transducer was approximately 44 Ω. The charging efficiency at different frequency is shown in Fig 15.

Subsequent tests fixed the acoustic transmission frequency at 44 kHz while varying the amplitude of the excitation voltage. When a peak-to-peak excitation voltage of 397 and a peak-to-peak current of 10.5 were used, the apparent electrical power input to the transmitting array was 521 W, while the electrical power delivered to the external sliding rheostat at the receiving end was 21.5 W, resulting in a transmission efficiency of 4.24%.

## Conclusions

Aiming at the demand for long-distance underwater acoustic wireless charging, this paper presents the design of a Composite rod-based transmitter-receiver array for underwater wireless power transfer. The electro-acoustic and acoustic-electric conversion mechanisms were analyzed using transmission line theory to determine key operational parameters for the transmitting and receiving transducer arrays, including operating frequency, bandwidth, transmitted power, and beamwidth. Furthermore, the design of a high-power, highly directional acoustic transducer array was investigated, encompassing the design of individual transducer elements and the overall array configuration. Correspondingly, the design of a resonant, large-aperture receiving transducer array was studied, focusing on its element structure and array layout. It should be noted that the transducer array elements in this study were manually assembled, and the inconsistent application of pre-stress caused performance deviations among the elements. Consequently, when these elements were integrated into the array, the overall performance of the array—including directivity, beamwidth, and sidelobe level—became inconsistent. Anechoic-water tank experiments demonstrated that the acoustic array achieved a wireless charging capability of 21.5 W with an efficiency of 4.24%.
