# Supplementary material for: Development and experimental validation of a composite rod underwater acoustic wireless charging system
Source: PLoS One. 2026 Aug 20;21(8):e0355236. doi: 10.1371/journal.pone.0355236 (PMC13492800; doi:10.1371/journal.pone.0355236)
Supplement: Supplementary file 3 [file pone.0355236.s003.docx]

- All the comments of the reviewers have been considered by the authors and the recommended corrections have been included well.
- The updated paper explains the experimental set up and transducer array configuration in a better way.
- The explanation of the deviation of resonance frequencies and the inability of manual assembly makes a great contribution to the technical understandability of the work.
- The additional description of the acoustic behavior at far and near fields enhances the scientific rationale of the experimental methodology.
- In the revised manuscript, there is a good consistency in simulation and experimental validation results.
- The figures, especially the new high-resolution Figure 5, have been made more qualified and clearer by the authors.
- The structure of the manuscript, the level of language and the presentation of the technical work have become better than before the revision.
- The introduction of updated data availability data and funding corrected information is suitable to the journal requirements.
- The work has significant experimental findings in the field of underwater acoustic wireless charging and is now technically viable.
- The revised version of the manuscript can be published and accepted.
