# Supplementary material for: Development and experimental validation of a composite rod underwater acoustic wireless charging system
Source: PLoS One. 2026 Aug 20;21(8):e0355236. doi: 10.1371/journal.pone.0355236 (PMC13492800; doi:10.1371/journal.pone.0355236)
Supplement: Supplementary file 4 [file pone.0355236.s001.docx]

1. The manuscript presents a well-designed and experimentally validated underwater acoustic wireless power transfer system, which is both timely and relevant.
2. The use of a composite rod piezoelectric transducer array is innovative and demonstrates a meaningful advancement in underwater energy transmission.
3. The authors provide comprehensive experimental validation, which strengthens the credibility of the proposed system.
4. The authors execute the integration of simulation and experimental results well, demonstrating good agreement.
5. The study addresses an important challenge in underwater wireless charging, particularly for marine and autonomous systems.
6. The design of the 9x9 transducer array with high directivity is technically sound and easy to understand.
7. The manuscript contributes useful insights into acoustic wave-based energy transfer mechanisms.
8. The results, although with modest efficiency, provide a solid foundation for future optimization and scaling.
9. The work is methodologically structured, with clear sections on design, fabrication, and testing.
10. With improvements in language, discussion, and comparative analysis, the manuscript is suitable for publication.
