# Supplementary material for: Development and experimental validation of a composite rod underwater acoustic wireless charging system
Source: PLoS One. 2026 Aug 20;21(8):e0355236. doi: 10.1371/journal.pone.0355236 (PMC13492800; doi:10.1371/journal.pone.0355236)
Supplement: Supplementary file 5 [file pone.0355236.s002.docx]

Dear Editor and Reviewers,

On behalf of my co-authors, I would like to express our sincere gratitude to the reviewers and editors for their constructive and insightful criticism and advice regarding our manuscript, " Development and Experimental Validation of a Composite Rod Underwater Acoustic Wireless Charging System (ID: PONE-D-26-06324)." The feedback provided is invaluable and has significantly contributed to the enhancement of our paper. Below, we present a point-by-point response to the reviewers' suggestions, highlighting the specific areas in the manuscript that have been modified. The accompanying documents include a tracked version that delineates all changes made, as well as a clean version of the revised manuscript. We once again express our sincere gratitude to the editors and reviewers for your hard work.

We wish you good health and great success in your work!

Sincerely,

Wei Sun

2026.04.04

**Journal Requirements:**

**1. Please ensure that your manuscript meets PLOS ONE's style requirements, including those for file naming.**

**Authors’ response:** We have revised the style of the article in accordance with the MANUSCRIPT BODY FORMATTING GUIDELINES and the revised manuscript with tracked changes is available.

**2. We note that the grant information you provided in the ‘Funding Information’ and ‘Financial Disclosure’ sections do not match.**

**When you resubmit, please ensure that you provide the correct grant numbers for the awards you received for your study in the ‘Funding Information’ section.**

**Authors’ response:** We thank the editor for pointing out this inconsistency. We have carefully reviewed the funding information and confirmed the correct grant numbers. The ‘Funding Information’ and ‘Financial Disclosure’ sections have now been unified, and the correct grant numbers are provided in the ‘Funding Information’ section. We apologize for the oversight and appreciate your attention to this matter.

**3 We note that your Data Availability Statement is currently as follows: [All relevant data are within the manuscript and its Supporting Information files.]**

**Authors’ response:** We thank the editor for this reminder. We have carefully reviewed the data availability requirements and have confirmed/updated our data availability statement as follows: All data underlying the findings in this study are publicly available in Figshare (<https://doi.org/>10.6084/m9.figshare.31932288). The dataset includes the raw values behind all reported graph and data points, and any extracted image data as required by PLOS ONE policy. No further data need to be uploaded.

**4 PLOS requires an ORCID iD for the corresponding author in Editorial Manager on papers submitted after December 6th, 2016. Please ensure that you have an ORCID iD and that it is validated in Editorial Manager. To do this, go to ‘Update my Information’ (in the upper left-hand corner of the main menu), and click on the Fetch/Validate link next to the ORCID field. This will take you to the ORCID site and allow you to create a new iD or authenticate a pre-existing iD in Editorial Manager.**

**Authors’ response:** Thank you for your reminder. We have authenticated a pre-existing iD in my personal information.

**5 Please ensure that you refer to Figure 1 in your text as, if accepted, production will need this reference to link the reader to the figure.**

**Authors’ response:** We thank the editor for this reminder. We have carefully reviewed the manuscript and confirmed that Figure 1 is now properly cited in the text (see line 43). All other figures have also been checked to ensure they are cited in the order of appearance.

**6. Please upload a new copy of Figure 5 as the detail is not clear.**

**Authors’ response:** We thank the editor for pointing this out. We have prepared a new, high-resolution version of Figure 5 with improved clarity and detail. The revised figure has been uploaded as a separate file in the submission system. We confirm that the content of the figure remains unchanged; only the resolution and quality have been enhanced to meet the journal’s requirements.

**7. We note that Figures 6, 8, 11, and 14 in your submission contain images which may be copyrighted. All PLOS content is published under the Creative Commons Attribution License (CC BY 4.0), which means that the manuscript, images, and Supporting Information files will be freely available online, and any third party is permitted to access, download, copy, distribute, and use these materials in any way, even commercially, with proper attribution. For more information, see our copyright guidelines:**

**Authors’ response:** We thank the editor for raising this copyright concern. Figures 6, 8, 11, and 14 are original works created by the authors and are not subject to third-party copyright restrictions. We confirm that these figures were generated during the course of this study and that all authors hold the rights to these images. Therefore, they can be published under the CC BY 4.0 license without restriction.

**Reviewer #1:**

**1.This is good work, but needs more details explaining the experimental setup and the sources of the deviation in the resonant frequencies in the modules. I'd like to know the system behavior at even smaller distances**

**Authors’ response:**

We thank the reviewer for the positive and constructive comments. We have added further details to the manuscript and address the two issues raised as follows.

**1. Source of resonance frequency deviation**

In this study, the transducer array elements were manually assembled. Due to the difficulty in applying exactly the same pre-stress to each element during assembly, certain deviations exist among the elements in terms of resonance frequency, electromechanical coupling coefficient, sensitivity, and other performance parameters. When these deviated elements are integrated into the array, the mutual coupling between elements and the superposition effect of the overall acoustic field amplify the aforementioned inconsistencies, ultimately leading to measurable deviations in the overall array performance, including directivity, beamwidth, and sidelobe level. We have added a discussion of limitations in the Conclusions section, explicitly stating that manual assembly is the main current limitation of this method.

Specific revised text (see line 250 to 255):

“It should be noted that the transducer array elements in this study were manually assembled, and the inconsistent application of pre-stress led to performance deviations among the elements. After the elements were integrated into the array, the overall performance of the array—including directivity, beamwidth, and sidelobe level—also became inconsistent.”

**2. System behavior at smaller distances**

We understand that the reviewer would like to know the behavior of the transducer at smaller transmitter–receiver distances (i.e., in the near-field region). According to acoustic theory, a transducer must operate under far-field conditions to accurately reflect its acoustic performance. The far-field criterion is given by:

$$d\geq\frac{a^{2}}{\lambda}$$

Where $d$ is the transmitter–receiver distance, $a$ is the maximum aperture of the transducer, and $\lambda$is the acoustic wavelength at the operating frequency.

When the receiver is located in the near-field region ($d<a^{2}/\lambda$), the acoustic field lies in the Fresnel interference zone. In this region, the superposition of acoustic waves is significant, and the sound pressure distribution oscillates drastically with both distance and lateral position, no longer following the spherical wave attenuation law valid under far-field conditions. Further reducing the distance does not monotonically improve the charging efficiency; on the contrary, due to destructive interference, the efficiency may exhibit local troughs and poor spatial repeatability. Consequently, efficiency measurements obtained in the near-field region cannot represent the true acoustic output capability of the transducer itself and are unsuitable for evaluating the performance of wireless power transfer systems.

Based on the physical mechanisms described above, this work conducts experiments at far-field distances to ensure the physical significance and reproducibility of the results. We have added this theoretical explanation to the revised manuscript to clarify why smaller distances were not tested and what behavior would be expected.

Specific revised text (see line 200 to 211):

“According to acoustic theory, a transducer must operate under far-field conditions to accurately reflect its acoustic performance. The far-field criterion is given by:

$$d\geq\frac{a^{2}}{\lambda}$$

Where d is the transmitter–receiver distance, a is the maximum aperture of the transducer, and \lambda is the acoustic wavelength at the operating frequency.

When the receiver is located in the near-field region ($d<a^{2}/\lambda$), the acoustic field lies in the Fresnel interference zone. In this region, the superposition of acoustic waves is significant, and the sound pressure distribution oscillates drastically with both distance and lateral position, no longer following the spherical wave attenuation law valid under far-field conditions. Further reducing the distance does not monotonically improve the charging efficiency; on the contrary, due to destructive interference, the efficiency may exhibit local troughs and poor spatial repeatability. Consequently, efficiency measurements obtained in the near-field region cannot represent the true acoustic output capability of the transducer itself and are unsuitable for evaluating the performance of wireless power transfer systems.

Based on the physical mechanisms described above, this work conducts experiments under far-field distances to ensure the physical significance and reproducibility of the results.”

**Reviewer #2:**

**1. The manuscript presents a well-designed and experimentally validated underwater acoustic wireless power transfer system, which is both timely and relevant.**

**2. The use of a composite rod piezoelectric transducer array is innovative and demonstrates a meaningful advancement in underwater energy transmission.**

**3. The authors provide comprehensive experimental validation, which strengthens the credibility of the proposed system.**

**4. The authors execute the integration of simulation and experimental results well, demonstrating good agreement.**

**5. The study addresses an important challenge in underwater wireless charging, particularly for marine and autonomous systems.**

**6. The design of the 9x9 transducer array with high directivity is technically sound and easy to understand.**

**7. The manuscript contributes useful insights into acoustic wave-based energy transfer mechanisms.**

**8. The results, although with modest efficiency, provide a solid foundation for future optimization and scaling.**

**9. The work is methodologically structured, with clear sections on design, fabrication, and testing.**

**10. With improvements in language, discussion, and comparative analysis, the manuscript is suitable for publication**

**Authors’ response:**

We sincerely thank the reviewer for the thorough and highly encouraging evaluation of our work. We are delighted that the reviewer recognizes the timeliness, innovation, experimental rigor, and methodological clarity of our manuscript. We also appreciate the reviewer’s constructive suggestions for further improvement. Below we address each comment in turn.

Comments 1–9:

We greatly appreciate the reviewer’s positive feedback on the overall design, experimental validation, simulation-experiment agreement, array architecture, and the foundational value of our results despite the current modest efficiency. These encouraging remarks motivate us to further advance this research.

Comment 10:

We thank the reviewer for the positive and constructive comments. We have added further details to the manuscript, as explained below.

Reason for module parameter deviations (see line 250 to 255): Because the transducer array elements were manually assembled, inconsistent application of pre-stress led to performance deviations among the elements. When these elements were integrated into the array, the overall performance of the array also became inconsistent.

Behavior at smaller distances (see line 200 to 211): The transducer must operate at far-field distances. The acoustic far-field criterion for a transducer is $d\geq a^{2}/\lambda$ (where $d$ is the transmitter–receiver distance, $a$ is the maximum aperture of the transducer, and $\lambda$ is the acoustic wavelength at the operating frequency). When the receiver is located in the near field (i.e., the distance is less than $a^{2}/\lambda$), the near-field region lies in the Fresnel interference zone, where acoustic waves superimpose severely and cannot truly reflect the acoustic performance of the transducer. Therefore, if charging experiments are conducted in the near-field region, the superposition of acoustic waves will instead lead to lower charging efficiency.
