# Supplementary material for: Development and experimental validation of a composite rod underwater acoustic wireless charging system
Source: PLoS One. 2026 Aug 20;21(8):e0355236. doi: 10.1371/journal.pone.0355236 (PMC13492800; doi:10.1371/journal.pone.0355236)
Supplement: Peer review history

# Peer review: Decision Letter 0

**DOI:** 10.1371/journal.pone.0355236.r001

**Authors:** Sushank Chaudhary

25 Mar 2026

Dear Dr. Sun,

Thank you for submitting your manuscript to PLOS ONE. After careful consideration, we feel that it has merit but does not fully meet PLOS ONE’s publication criteria as it currently stands. Therefore, we invite you to submit a revised version of the manuscript that addresses the points raised during the review process.

Please submit your revised manuscript by May 09 2026 11:59PM. If you will need more time than this to complete your revisions, please reply to this message or contact the journal office at plosone@plos.org. When you're ready to submit your revision, log on to https://www.editorialmanager.com/pone/ and select the 'Submissions Needing Revision' folder to locate your manuscript file.

- A letter that responds to each point raised by the academic editor and reviewer(s). You should upload this letter as a separate file labeled 'Response to Reviewers'.
- A marked-up copy of your manuscript that highlights changes made to the original version. You should upload this as a separate file labeled 'Revised Manuscript with Track Changes'.
- An unmarked version of your revised paper without tracked changes. You should upload this as a separate file labeled 'Manuscript'.

If you would like to make changes to your financial disclosure, please include your updated statement in your cover letter. Guidelines for resubmitting your figure files are available below the reviewer comments at the end of this letter.

If applicable, we recommend that you deposit your laboratory protocols in protocols.io to enhance the reproducibility of your results. Protocols.io assigns your protocol its own identifier (DOI) so that it can be cited independently in the future. For instructions see: https://journals.plos.org/plosone/s/submission-guidelines#loc-laboratory-protocols. Additionally, PLOS ONE offers an option for publishing peer-reviewed Lab Protocol articles, which describe protocols hosted on protocols.io. Read more information on sharing protocols at https://plos.org/protocols?utm_medium=editorial-email&utm_source=authorletters&utm_campaign=protocols.

As the corresponding author, your ORCID iD is verified in the submission system and will appear in the published article. PLOS supports the use of ORCID, and we encourage all coauthors to register for an ORCID iD and use it as well. Please encourage your coauthors to verify their ORCID iD within the submission system before final acceptance, as unverified ORCID iDs will not appear in the published article. *Only*  the individual author can complete the verification step; PLOS staff *cannot*  verify ORCID iDs on behalf of authors.

We look forward to receiving your revised manuscript.

Kind regards,

Sushank Chaudhary, Ph.D

Academic Editor

PLOS One

Journal Requirements:

When submitting your revision, we need you to address these additional requirements.

1. Please ensure that your manuscript meets PLOS ONE's style requirements, including those for file naming. The PLOS ONE style templates can be found at

https://journals.plos.org/plosone/s/file?id=wjVg/PLOSOne_formatting_sample_main_body.pdf and https://journals.plos.org/plosone/s/file?id=ba62/PLOSOne_formatting_sample_title_authors_affiliations.pdf

2. Please note that PLOS One has specific guidelines on code sharing for submissions in which author-generated code underpins the findings in the manuscript. In these cases, we expect all author-generated code to be made available without restrictions upon publication of the work. Please review our guidelines at https://journals.plos.org/plosone/s/materials-and-software-sharing#loc-sharing-code and ensure that your code is shared in a way that follows best practice and facilitates reproducibility and reuse.

3. We note that the grant information you provided in the ‘Funding Information’ and ‘Financial Disclosure’ sections do not match.

When you resubmit, please ensure that you provide the correct grant numbers for the awards you received for your study in the ‘Funding Information’ section.

4. Thank you for stating the following financial disclosure:

[The author(s) received no specific funding for this work.].

At this time, please address the following queries:

a) Please clarify the sources of funding (financial or material support) for your study. List the grants or organizations that supported your study, including funding received from your institution.

b) State what role the funders took in the study. If the funders had no role in your study, please state: “The funders had no role in study design, data collection and analysis, decision to publish, or preparation of the manuscript.”

c) If any authors received a salary from any of your funders, please state which authors and which funders.

d) If you did not receive any funding for this study, please state: “The authors received no specific funding for this work.”

Please include your amended statements within your cover letter; we will change the online submission form on your behalf.

5. Thank you for stating the following in the Acknowledgments Section of your manuscript:

[This work was supported by the Programs for Shandong Key R&D Plan (Grant No. ZFJH202305-2)]

We note that you have provided funding information that is not currently declared in your Funding Statement. However, funding information should not appear in the Acknowledgments section or other areas of your manuscript. We will only publish funding information present in the Funding Statement section of the online submission form.

Please remove any funding-related text from the manuscript and let us know how you would like to update your Funding Statement. Currently, your Funding Statement reads as follows:

[The author(s) received no specific funding for this work.]

Please include your amended statements within your cover letter; we will change the online submission form on your behalf.

6. We note that your Data Availability Statement is currently as follows: [All relevant data are within the manuscript and its Supporting Information files.]

Please confirm at this time whether or not your submission contains all raw data required to replicate the results of your study. Authors must share the “minimal data set” for their submission. PLOS defines the minimal data set to consist of the data required to replicate all study findings reported in the article, as well as related metadata and methods (https://journals.plos.org/plosone/s/data-availability#loc-minimal-data-set-definition).

For example, authors should submit the following data:

- The values behind the means, standard deviations and other measures reported;

- The values used to build graphs;

- The points extracted from images for analysis.

Authors do not need to submit their entire data set if only a portion of the data was used in the reported study.

If your submission does not contain these data, please either upload them as Supporting Information files or deposit them to a stable, public repository and provide us with the relevant URLs, DOIs, or accession numbers. For a list of recommended repositories, please see https://journals.plos.org/plosone/s/recommended-repositories.

If there are ethical or legal restrictions on sharing a de-identified data set, please explain them in detail (e.g., data contain potentially sensitive information, data are owned by a third-party organization, etc.) and who has imposed them (e.g., an ethics committee). Please also provide contact information for a data access committee, ethics committee, or other institutional body to which data requests may be sent. If data are owned by a third party, please indicate how others may request data access.

7. PLOS requires an ORCID iD for the corresponding author in Editorial Manager on papers submitted after December 6th, 2016. Please ensure that you have an ORCID iD and that it is validated in Editorial Manager. To do this, go to ‘Update my Information’ (in the upper left-hand corner of the main menu), and click on the Fetch/Validate link next to the ORCID field. This will take you to the ORCID site and allow you to create a new iD or authenticate a pre-existing iD in Editorial Manager.

8. Please ensure that you refer to Figure 1 in your text as, if accepted, production will need this reference to link the reader to the figure.

9. Please upload a new copy of Figure 5 as the detail is not clear. Please follow the link for more information:  https://journals.plos.org/plosone/s/figures

10. We note that Figures 6, 8, 11, and 14 in your submission contain images which may be copyrighted. All PLOS content is published under the Creative Commons Attribution License (CC BY 4.0), which means that the manuscript, images, and Supporting Information files will be freely available online, and any third party is permitted to access, download, copy, distribute, and use these materials in any way, even commercially, with proper attribution. For more information, see our copyright guidelines: http://journals.plos.org/plosone/s/licenses-and-copyright.

We require you to either (1) present written permission from the copyright holder to publish these figures specifically under the CC BY 4.0 license, or (2) remove the figures from your submission:

1. You may seek permission from the original copyright holder of Figures 6, 8, 11, and 14 to publish the content specifically under the CC BY 4.0 license.

We recommend that you contact the original copyright holder with the Content Permission Form (http://journals.plos.org/plosone/s/file?id=7c09/content-permission-form.pdf) and the following text:

“I request permission for the open-access journal PLOS ONE to publish XXX under the Creative Commons Attribution License (CCAL) CC BY 4.0 (http://creativecommons.org/licenses/by/4.0/). Please be aware that this license allows unrestricted use and distribution, even commercially, by third parties. Please reply and provide explicit written permission to publish XXX under a CC BY license and complete the attached form.”

Please upload the completed Content Permission Form or other proof of granted permissions as an "Other" file with your submission.

In the figure caption of the copyrighted figure, please include the following text: “Reprinted from [ref] under a CC BY license, with permission from [name of publisher], original copyright [original copyright year].”

2. If you are unable to obtain permission from the original copyright holder to publish these figures under the CC BY 4.0 license or if the copyright holder’s requirements are incompatible with the CC BY 4.0 license, please either i) remove the figure or ii) supply a replacement figure that complies with the CC BY 4.0 license. Please check copyright information on all replacement figures and update the figure caption with source information. If applicable, please specify in the figure caption text when a figure is similar but not identical to the original image and is therefore for illustrative purposes only.

If the reviewer comments include a recommendation to cite specific previously published works, please review and evaluate these publications to determine whether they are relevant and should be cited. There is no requirement to cite these works unless the editor has indicated otherwise.

[Note: HTML markup is below. Please do not edit.]

Reviewers' comments:

Reviewer's Responses to Questions

**Comments to the Author**

1. Is the manuscript technically sound, and do the data support the conclusions?

Reviewer #1: Yes

Reviewer #2: Yes

**********

2. Has the statistical analysis been performed appropriately and rigorously?

Reviewer #1: I Don't Know

Reviewer #2: Yes

**********

3. Have the authors made all data underlying the findings in their manuscript fully available?

The PLOS Data policy

Reviewer #1: No

Reviewer #2: Yes

**********

4. Is the manuscript presented in an intelligible fashion and written in standard English?

Reviewer #1: Yes

Reviewer #2: Yes

**********

Reviewer #1: This is good work, but needs more details explaining the experimental setup and the sources of the deviation in the resonant frequencies in the modules. I'd like to know the system behavior at even smaller distances.

Reviewer #2: 1. The manuscript presents a well-designed and experimentally validated underwater acoustic wireless power transfer system, which is both timely and relevant.

2. The use of a composite rod piezoelectric transducer array is innovative and demonstrates a meaningful advancement in underwater energy transmission.

3. The authors provide comprehensive experimental validation, which strengthens the credibility of the proposed system.

4. The authors execute the integration of simulation and experimental results well, demonstrating good agreement.

5. The study addresses an important challenge in underwater wireless charging, particularly for marine and autonomous systems.

6. The design of the 9x9 transducer array with high directivity is technically sound and easy to understand.

7. The manuscript contributes useful insights into acoustic wave-based energy transfer mechanisms.

8. The results, although with modest efficiency, provide a solid foundation for future optimization and scaling.

9. The work is methodologically structured, with clear sections on design, fabrication, and testing.

10. With improvements in language, discussion, and comparative analysis, the manuscript is suitable for publication.

**********

what does this mean?). If published, this will include your full peer review and any attached files.

If you choose “no”, your identity will remain anonymous but your review may still be made public.

**Do you want your identity to be public for this peer review?**  For information about this choice, including consent withdrawal, please see our Privacy Policy

Reviewer #1: **Yes:**  Christian Mai

Reviewer #2: **Yes:**  Gurumurthy BR

**********

[NOTE: If reviewer comments were submitted as an attachment file, they will be attached to this email and accessible via the submission site. Please log into your account, locate the manuscript record, and check for the action link "View Attachments". If this link does not appear, there are no attachment files.]

To ensure your figures meet our technical requirements, please review our figure guidelines: https://journals.plos.org/plosone/s/figures

You may also use PLOS’s free figure tool, NAAS, to help you prepare publication quality figures: https://journals.plos.org/plosone/s/figures#loc-tools-for-figure-preparation.

NAAS will assess whether your figures meet our technical requirements by comparing each figure against our figure specifications.

Attachment Submitted filename: Gurumurthy BR.docx

# Author comment: Author response to Decision Letter 1

**DOI:** 10.1371/journal.pone.0355236.r002

11 Apr 2026

Dear Editor and Reviewers,

On behalf of my co-authors, I would like to express our sincere gratitude to the reviewers and editors for their constructive and insightful criticism and advice regarding our manuscript, " Development and Experimental Validation of a Composite Rod Underwater Acoustic Wireless Charging System (ID: PONE-D-26-06324)." The feedback provided is invaluable and has significantly contributed to the enhancement of our paper. Below, we present a point-by-point response to the reviewers' suggestions, highlighting the specific areas in the manuscript that have been modified. The accompanying documents include a tracked version that delineates all changes made, as well as a clean version of the revised manuscript. We once again express our sincere gratitude to the editors and reviewers for your hard work.

We wish you good health and great success in your work!

Sincerely,

Wei Sun

2026.04.04

Journal Requirements:

1. Please ensure that your manuscript meets PLOS ONE's style requirements, including those for file naming.

Authors’ response: We have revised the style of the article in accordance with the MANUSCRIPT BODY FORMATTING GUIDELINES and the revised manuscript with tracked changes is available.

2. We note that the grant information you provided in the ‘Funding Information’ and ‘Financial Disclosure’ sections do not match.

When you resubmit, please ensure that you provide the correct grant numbers for the awards you received for your study in the ‘Funding Information’ section.

Authors’ response: We thank the editor for pointing out this inconsistency. We have carefully reviewed the funding information and confirmed the correct grant numbers. The ‘Funding Information’ and ‘Financial Disclosure’ sections have now been unified, and the correct grant numbers are provided in the ‘Funding Information’ section. We apologize for the oversight and appreciate your attention to this matter.

3 We note that your Data Availability Statement is currently as follows: [All relevant data are within the manuscript and its Supporting Information files.]

Authors’ response: We thank the editor for this reminder. We have carefully reviewed the data availability requirements and have confirmed/updated our data availability statement as follows: All data underlying the findings in this study are publicly available in Figshare (https://doi.org/10.6084/m9.figshare.31932288).

4 PLOS requires an ORCID iD for the corresponding author in Editorial Manager on papers submitted after December 6th, 2016. Please ensure that you have an ORCID iD and that it is validated in Editorial Manager. To do this, go to ‘Update my Information’ (in the upper left-hand corner of the main menu), and click on the Fetch/Validate link next to the ORCID field. This will take you to the ORCID site and allow you to create a new iD or authenticate a pre-existing iD in Editorial Manager.

Authors’ response: Thank you for your reminder. We have authenticated a pre-existing iD in my personal information.

5 Please ensure that you refer to Figure 1 in your text as, if accepted, production will need this reference to link the reader to the figure.

Authors’ response: We thank the editor for this reminder. We have carefully reviewed the manuscript and confirmed that Figure 1 is now properly cited in the text (see line 43). All other figures have also been checked to ensure they are cited in the order of appearance.

6. Please upload a new copy of Figure 5 as the detail is not clear.

Authors’ response: We thank the editor for pointing this out. We have prepared a new, high-resolution version of Figure 5 with improved clarity and detail. The revised figure has been uploaded as a separate file in the submission system. We confirm that the content of the figure remains unchanged; only the resolution and quality have been enhanced to meet the journal’s requirements.

7. We note that Figures 6, 8, 11, and 14 in your submission contain images which may be copyrighted. All PLOS content is published under the Creative Commons Attribution License (CC BY 4.0), which means that the manuscript, images, and Supporting Information files will be freely available online, and any third party is permitted to access, download, copy, distribute, and use these materials in any way, even commercially, with proper attribution. For more information, see our copyright guidelines:

Authors’ response: We thank the editor for raising this copyright concern. Figures 6, 8, 11, and 14 are original works created by the authors and are not subject to third-party copyright restrictions. We confirm that these figures were generated during the course of this study and that all authors hold the rights to these images. Therefore, they can be published under the CC BY 4.0 license without restriction.

Reviewer #1:

1.This is good work, but needs more details explaining the experimental setup and the sources of the deviation in the resonant frequencies in the modules. I'd like to know the system behavior at even smaller distances

Authors’ response:

We thank the reviewer for the positive and constructive comments. We have added further details to the manuscript and address the two issues raised as follows.

1. Source of resonance frequency deviation

In this study, the transducer array elements were manually assembled. Due to the difficulty in applying exactly the same pre-stress to each element during assembly, certain deviations exist among the elements in terms of resonance frequency, electromechanical coupling coefficient, sensitivity, and other performance parameters. When these deviated elements are integrated into the array, the mutual coupling between elements and the superposition effect of the overall acoustic field amplify the aforementioned inconsistencies, ultimately leading to measurable deviations in the overall array performance, including directivity, beamwidth, and sidelobe level. We have added a discussion of limitations in the Conclusions section, explicitly stating that manual assembly is the main current limitation of this method.

Specific revised text (see line 250 to 255):

“It should be noted that the transducer array elements in this study were manually assembled, and the inconsistent application of pre-stress led to performance deviations among the elements. After the elements were integrated into the array, the overall performance of the array—including directivity, beamwidth, and sidelobe level—also became inconsistent.”

2. System behavior at smaller distances

We understand that the reviewer would like to know the behavior of the transducer at smaller transmitter–receiver distances (i.e., in the near-field region). According to acoustic theory, a transducer must operate under far-field conditions to accurately reflect its acoustic performance. The far-field criterion is given by:

d\geq\frac{a^2}{\lambda}

Where d is the transmitter–receiver distance, a is the maximum aperture of the transducer, and \lambda\ is the acoustic wavelength at the operating frequency.

When the receiver is located in the near-field region (d<a^2/\lambda), the acoustic field lies in the Fresnel interference zone. In this region, the superposition of acoustic waves is significant, and the sound pressure distribution oscillates drastically with both distance and lateral position, no longer following the spherical wave attenuation law valid under far-field conditions. Further reducing the distance does not monotonically improve the charging efficiency; on the contrary, due to destructive interference, the efficiency may exhibit local troughs and poor spatial repeatability. Consequently, efficiency measurements obtained in the near-field region cannot represent the true acoustic output capability of the transducer itself and are unsuitable for evaluating the performance of wireless power transfer systems.

Based on the physical mechanisms described above, this work conducts experiments at far-field distances to ensure the physical significance and reproducibility of the results. We have added this theoretical explanation to the revised manuscript to clarify why smaller distances were not tested and what behavior would be expected.

Specific revised text (see line 200 to 211):

“According to acoustic theory, a transducer must operate under far-field conditions to accurately reflect its acoustic performance. The far-field criterion is given by:

d\geq\frac{a^2}{\lambda}

Where d is the transmitter–receiver distance, a is the maximum aperture of the transducer, and \lambda is the acoustic wavelength at the operating frequency.

When the receiver is located in the near-field region (d<a^2/\lambda), the acoustic field lies in the Fresnel interference zone. In this region, the superposition of acoustic waves is significant, and the sound pressure distribution oscillates drastically with both distance and lateral position, no longer following the spherical wave attenuation law valid under far-field conditions. Further reducing the distance does not monotonically improve the charging efficiency; on the contrary, due to destructive interference, the efficiency may exhibit local troughs and poor spatial repeatability. Consequently, efficiency measurements obtained in the near-field region cannot represent the true acoustic output capability of the transducer itself and are unsuitable for evaluating the performance of wireless power transfer systems.

Based on the physical mechanisms described above, this work conducts experiments under far-field distances to ensure the physical significance and reproducibility of the results.”

Reviewer #2:

1. The manuscript presents a well-designed and experimentally validated underwater acoustic wireless power transfer system, which is both timely and relevant.

2. The use of a composite rod piezoelectric transducer array is innovative and demonstrates a meaningful advancement in underwater energy transmission.

3. The authors provide comprehensive experimental validation, which strengthens the credibility of the proposed system.

4. The authors execute the integration of simulation and experimental results well, demonstrating good agreement.

5. The study addresses an important challenge in underwater wireless charging, particularly for marine and autonomous systems.

6. The design of the 9x9 transducer array with high directivity is technically sound and easy to understand.

7. The manuscript contributes useful insights into acoustic wave-based energy transfer mechanisms.

8. The results, although with modest efficiency, provide a solid foundation for future optimization and scaling.

9. The work is methodologically structured, with clear sections on design, fabrication, and testing.

10. With improvements in language, discussion, and comparative analysis, the manuscript is suitable for publication

Authors’ response:

We sincerely thank the reviewer for the thorough and highly encouraging evaluation of our work. We are delighted that the reviewer recognizes the timeliness, innovation, experimental rigor, and methodological clarity of our manuscript. We also appreciate the reviewer’s constructive suggestions for further improvement. Below we address each comment in turn.

Comments 1–9:

We greatly appreciate the reviewer’s positive feedback on the overall design, experimental validation, simulation-experiment agreement, array architecture, and the foundational value of our results despite the current modest efficiency. These encouraging remarks motivate us to further advance this research.

Comment 10:

We thank the reviewer for the positive and constructive comments. We have added further details to the manuscript, as explained below.

Reason for module parameter deviations (see line 250 to 255): Because the transducer array elements were manually assembled, inconsistent application of pre-stress led to performance deviations among the elements. When these elements were integrated into the array, the overall performance of the array also became inconsistent.

Behavior at smaller distances (see line 200 to 211): The transducer must operate at far-field distances. The acoustic far-field criterion for a transducer is d\geq a^2/\lambda (where d is the transmitter–receiver distance, a is the maximum aperture of the transducer, and \lambda is the acoustic wavelength at the operating frequency). When the receiver is located in the near field (i.e., the distance is less than a^2/\lambda), the near-field region lies in the Fresnel interference zone, where acoustic waves superimpose severely and cannot truly reflect the acoustic performance of the transducer. Therefore, if charging experiments are conducted in the near-field region, the superposition of acoustic waves will instead lead to lower charging efficiency.

Attachment Submitted filename: Response to Reviewers.docx

# Peer review: Decision Letter 1

**DOI:** 10.1371/journal.pone.0355236.r003

**Authors:** Sushank Chaudhary

19 Jul 2026

<p>Development and Experimental Validation of a Composite Rod Underwater Acoustic Wireless Charging System

PONE-D-26-06324R1

Dear Dr. Sun,

We’re pleased to inform you that your manuscript has been judged scientifically suitable for publication and will be formally accepted for publication once it meets all outstanding technical requirements.

Within one week, you’ll receive an e-mail detailing the required amendments. When these have been addressed, you’ll receive a formal acceptance letter and your manuscript will be scheduled for publication.

An invoice will be generated when your article is formally accepted. Please note, if your institution has a publishing partnership with PLOS and your article meets the relevant criteria, all or part of your publication costs will be covered. Please make sure your user information is up-to-date by logging into Editorial Manager at Editorial Manager® and clicking the ‘Update My Information' link at the top of the page. For questions related to billing, please contact billing support.

If your institution or institutions have a press office, please notify them about your upcoming paper to help maximize its impact. If they’ll be preparing press materials, please inform our press team as soon as possible -- no later than 48 hours after receiving the formal acceptance. Your manuscript will remain under strict press embargo until 2 pm Eastern Time on the date of publication. For more information, please contact onepress@plos.org.

Kind regards,

Sushank Chaudhary, Ph.D

Academic Editor

PLOS One

Additional Editor Comments (optional):

Reviewers' comments:

Reviewer's Responses to Questions

**Comments to the Author**

Reviewer #2: All comments have been addressed

**********

2. Is the manuscript technically sound, and do the data support the conclusions?

Reviewer #2: Yes

**********

3. Has the statistical analysis been performed appropriately and rigorously?

Reviewer #2: N/A

**********

4. Have the authors made all data underlying the findings in their manuscript fully available?

The PLOS Data policy

Reviewer #2: Yes

**********

5. Is the manuscript presented in an intelligible fashion and written in standard English?

Reviewer #2: Yes

**********

Reviewer #2: • All the comments of the reviewers have been considered by the authors and the recommended corrections have been included well.

• The updated paper explains the experimental set up and transducer array configuration in a better way.

• The explanation of the deviation of resonance frequencies and the inability of manual assembly makes a great contribution to the technical understandability of the work.

• The additional description of the acoustic behavior at far and near fields enhances the scientific rationale of the experimental methodology.

• In the revised manuscript, there is a good consistency in simulation and experimental validation results.

• The figures, especially the new high-resolution Figure 5, have been made more qualified and clearer by the authors.

• The structure of the manuscript, the level of language and the presentation of the technical work have become better than before the revision.

• The introduction of updated data availability data and funding corrected information is suitable to the journal requirements.

• The work has significant experimental findings in the field of underwater acoustic wireless charging and is now technically viable.

• The revised version of the manuscript can be published and accepted.

**********

what does this mean?). If published, this will include your full peer review and any attached files.

If you choose “no”, your identity will remain anonymous but your review may still be made public.

**Do you want your identity to be public for this peer review?**  For information about this choice, including consent withdrawal, please see our Privacy Policy

Reviewer #2: **Yes:**  Gurumurthy BR

**********

Attachment Submitted filename: Gurumurthy B.R.docx

# Editor report: Acceptance letter

**DOI:** 10.1371/journal.pone.0355236.r004

**Authors:** Sushank Chaudhary

PONE-D-26-06324R1

PLOS One

Dear Dr. Sun,

I'm pleased to inform you that your manuscript has been deemed suitable for publication in PLOS One. Congratulations! Your manuscript is now being handed over to our production team.

At this stage, our production department will prepare your paper for publication. This includes ensuring the following:

* All references, tables, and figures are properly cited

* All relevant supporting information is included in the manuscript submission,

* There are no issues that prevent the paper from being properly typeset

You will receive further instructions from the production team, including instructions on how to review your proof when it is ready. Please keep in mind that we are working through a large volume of accepted articles, so please give us a few days to review your paper and let you know the next and final steps.

Lastly, if your institution or institutions have a press office, please let them know about your upcoming paper now to help maximize its impact. If they'll be preparing press materials, please inform our press team within the next 48 hours. Your manuscript will remain under strict press embargo until 2 pm Eastern Time on the date of publication. For more information, please contact onepress@plos.org.

You will receive an invoice from PLOS for your publication fee after your manuscript has reached the completed accept phase. If you receive an email requesting payment before acceptance or for any other service, this may be a phishing scheme. Learn how to identify phishing emails and protect your accounts at https://explore.plos.org/phishing.

If we can help with anything else, please email us at customercare@plos.org.

Thank you for submitting your work to PLOS ONE and supporting open access.

Kind regards,

PLOS ONE Editorial Office Staff

on behalf of

Prof. Sushank Chaudhary

Academic Editor

PLOS One
